# Independent Encoding of Orientation and Mean Luminance by Mouse Visual Cortex

**DOI:** 10.1523/ENEURO.0281-25.2025

**Published:** 2026-02-06

**Authors:** Ronan T. O’Shea, Xue-Xin Wei, Nicholas J. Priebe

**Affiliations:** ^1^ Center for Perceptual Systems, The University of Texas at Austin, Austin, Texas 78712; ^2^ Department of Psychology, The University of Texas at Austin, Austin, Texas 78712; ^3^ Center for Learning and Memory, The University of Texas at Austin, Austin, Texas 78712; ^4^Department of Neuroscience, The University of Texas at Austin, Austin, Texas 78712

**Keywords:** luminance, neocortex, population code

## Abstract

Natural environments contain behaviorally relevant information along many stimulus dimensions, each of which sensory systems must encode in order to guide behaviors. For example, the mammalian visual cortex encodes features of visual scenes such as spatial information related to object identity and temporal information about the motion of those objects in space. In order to reliably encode these behaviorally relevant visual features, neural representations should be robust to changes in environmental conditions. Further, information about changes in environmental conditions, such as the luminance changes that occur over the course of a day, is also important for guiding behaviors. In this study, we asked whether mouse primary visual cortex (V1) jointly represents the spatial properties of visual stimuli along with changes in the mean luminance of the visual scene. We find that while V1 neurons, in mice of either sex, encode spatial aspects of visual information in an invariant manner across luminance conditions, the V1 population response also contains a robust representation of luminance. Importantly, V1 populations encode changes in stimulus orientation and mean luminance along orthogonal axes in the neural response space, such that a change in one stimulus variable is encoded independently from the other.

## Significance Statement

We recorded from neural populations in mouse V1 with two-photon imaging to examine how sensory information along multiple feature axes is distributed across the responses of diversely tuned neurons. We find that the V1 population response contains a representation of mean luminance in addition to maintaining a luminance-invariant spatial representation. These independent representations are possible because stimulus information is distributed randomly across the V1 population, such that changes in each stimulus variable are encoded along orthogonal axes in the neural response space. This study offers an example of how multidimensional sensory representations emerge from the diverse response properties of neocortical neurons.

## Introduction

Sensory systems must simultaneously encode diverse features of environments in order to guide behaviors. To identify objects in a visual scene, the visual system initially transduces the spatiotemporal properties of visual inputs. But those signals will vary both as a function of the objects present in a scene as well as with the coincident luminance conditions. Identifying an object therefore requires disentangling environmental luminance signals from object properties. And yet it is also the case that luminance conditions provide behaviorally relevant information about time of day, weather conditions, and season. Environmental luminance can vary independently from the spatial properties of objects, and this fact is essential for guiding visual behaviors across environmental states. For example, the visual system should be able to recognize the same object whether illuminated by midday sun or moonlight, although the appropriate behavioral response may depend on the present environmental conditions. This suggests that an important function of the visual system is to independently encode spatial features and environmental luminance. It has been unclear how the visual system jointly encodes the spatial features and mean luminance of visual scenes.

The peripheral visual system adapts to changes in mean luminance in order to retain sensitivity and avoid saturation as light levels vary over 10 orders of magnitude between midnight and midday. Changes in pupil diameter, shifts in the sensitivity to photon absorption within a photoreceptor, and the transition between rod and cone phototransduction each help to maintain visual sensitivity across the range of environmental luminance intensities (Shapley and Enroth-Cugell, 1984; Nikonov et al., 1998; Field and Chichilnisky, 2007). The net effect of these complementary mechanisms is a shift in the operating point of the retinal ganglion cells (RGCs), which pool inputs from photoreceptors and project to central visual regions of the brain. In this framework, it is not apparent how RGC activity could encode mean luminance level, given that an aim of these adaptation mechanisms is to maintain invariant visual response properties within a narrow range of co-occurring luminance levels rather than encoding the larger changes in mean luminance that occur slowly over the course of a day.

There are several mechanisms by which central visual regions could encode the luminance state of the environment. One possibility is that V1 responses carry luminance information implicitly through tuning shifts that accompany luminance adaptation. In species with a foveated retina, tuning to high spatial frequencies in the center of the visual field is lost at scotopic luminance levels when inputs from densely packed cones are absent (Wikler et al., 1990). In addition, the spectral information present in the retinal code, which gives rise to color-opponent encoding under photopic conditions, is absent in the visual signal relayed by monochromatic rods under scotopic conditions (Baylor et al., 1987). Prior work has revealed shifts in chromatic contrast tuning in V1 neurons as a function of mean luminance (Rhim et al., 2017, 2021; Rhim and Nauhaus, 2023). Since these shifts in tuning are tied to mean luminance, these changes in V1 response properties are examples of implicit encoding of luminance adaptation state.

Visual areas may also have access to an explicit representation of mean luminance. Rodents and humans can discriminate the brightness of visual inputs in the absence of changes in rod and cone phototransduction (Brown et al., 2012). This work suggests that mammals have perceptual access to inputs from intrinsically photosensitive RGCs (ipRGCs), which encode the absolute luminance of visual inputs in a temporally low-pass manner (Do and Yau, 2010).

Prior work has shown that the visual responses in mouse V1 to monochromatic stimuli are invariant to scotopic versus photopic luminance, despite the functional shifts in the RGC population response across adaptation states (O’Shea et al., 2025). This study found no systematic shifts in the spatiotemporal tuning or correlation structure of the V1 population response between luminance levels. This study controls for the shifts in chromatic tuning which could implicitly encode environmental luminance by restricting stimuli to the overlapping chromatic sensitivity of mouse rods and M-cones (Rhim et al., 2021).

In the present study, we asked whether, in addition to the invariant spatiotemporal representation of visual features, the mean luminance level was encoded by the V1 population. We find that the V1 population response to spatiotemporally identical stimuli reliably encodes scotopic versus photopic luminance levels. Information about changes in visual orientation and luminance is distributed randomly across the V1 population. An important feature of this encoding scheme is that changes in orientation and luminance are encoded along orthogonal axes in the neural response space. We show that the independent encoding of orientation and luminance differs from the joint encoding of other stimulus features, such as spatial and temporal frequency (TF). These results show how multiple stimulus variables can be simultaneously represented by neural populations in the sensory cortex, in a manner that preserves the independent representations of these variables.

## Materials and Methods

### Animal subjects

All animal procedures were approved by The University of Texas at Austin Institutional Animal Care and Use Committee.

Imaging experiments were conducted using adult Ai94(TITL-GCaMP6s)-D;CaMK2a-tTA (The Jackson Laboratory #024115) mice of both sexes which express fluorescent calcium indicator GCaMP6s in forebrain excitatory neurons (*n* = 15). All mice were 6 weeks of age or older.

### Surgery

For all surgical procedures, mice were anesthetized with isoflurane (2.5% induction, 1–1.5% surgery) and given two preoperative subcutaneous injections of analgesia (5 mg/kg carprofen, 3.5 mg/kg Ethiqa) and an anti-inflammatory agent (dexamethasone, 1%). Mice were kept warm with a water-regulated pump pad. Each mouse underwent two surgical procedures. First, we placed a metal frame over the visual cortex using dental acrylic to be used for head fixation of the mouse during subsequent experiments. Second, we drilled a 1−4 mm craniotomy over V1 in the right hemisphere and sealed it with a glass window implant. Surgical procedures were always performed on a separate day from experiments. Following head frame implantation, we mapped the V1 retinotopy with intrinsic signal imaging as described previously (Juavinett et al., 2017; Rhim et al., 2017). In all experiments, we targeted the lower visual field of V1 based on the retinotopic map acquired with intrinsic signal imaging, in order to record from neurons which strongly respond to 525 nm stimuli at scotopic and photopic luminance levels (Rhim et al., 2017).

### Imaging

Two-photon calcium imaging was performed with a Neurolabware microscope and ScanBox acquisition software. The scan rate varied between 10 and 15 frames/s, scaling with the number of rows in the field of view. A Chameleon Ultra laser was set to 920 nm to excite GCaMP6s. A Nikon 16× (0.8 NA 3 mm WD) or Olympus 10× (0.6 NA 3 mm WD) objective lens was used for all imaging sessions, with 900- and 1,400-µm-wide field of view, respectively. All cells were imaged between 150 and 350 µm depth, which corresponds to layer 2/3 in the mouse. The average power of the exposed laser beam while imaging was approximately 60 and 30 mW for the 10× and 16× objectives, respectively.

Putative cells were extracted from our imaging data using the suite2p pipeline in Python (Pachitariu et al., 2017). We performed additional processing using custom MATLAB code to retain only cells meeting certain criteria. Cell fluorescent traces had to have skewness greater than 2. Cells also had to show significant visually evoked responses above baseline in their trial-averaged time courses as quantified by a one-sided, two-sample *t* test with *p* < 0.001. Finally, only cells that were identified and retained for both scotopic and photopic conditions were analyzed. Cells were matched across conditions by finding ROIs matched in both location and morphology, using custom MATLAB code.

### Stimulus presentation

#### Setup

A monochrome LED projector by Texas Instruments (Keynote Photonics) with a spectral peak at 525 nm was used to generate stimuli with a 60 Hz refresh rate onto a Teflon screen which provides a near-Lambertian surface (Rhim et al., 2017). The screen was 12.5 cm high × 32 cm wide, equating to approximately 64° × 116° of visual angle. Stimuli were coded using the Psychophysics Toolbox extension in MATLAB (Rhim et al., 2021). Mice were positioned such that the perpendicular bisector from the mouse's eye to the screen was 10 cm with the screen angled at 30° from the mouse's midline. The upper edge of the screen was placed near the vertical midline of the mouse's field of view to target the lower visual field for stimulation.

All visual stimuli were presented in the lower visual field (anterior V1). The anterior portion of V1 receives retinal input from M-opsin expressing cones under photopic conditions which have a similar chromatic response profile to rods in the mouse retina (Wang et al., 2011; Chang et al., 2013; Rhim et al., 2017, 2021). The similarity in chromatic response profiles allows us to use the same chromatic stimulus (525 nm) for both scotopic and photopic light levels, varying only the luminance of the stimulus.

#### Natural movies

We presented two, 10-s-long natural movies with each repeat followed by a 6-s-long blank screen at mean luminance. Movie 1 shows honeybees flying in a garden (by courtesy of Ian Nauhaus, The University of Texas at Austin), and Movie 2 shows monkeys playing in snow (by courtesy of David Leopold, NIMH). 

#### Gratings

In experiments comparing V1 responses under scotopic and photopic luminance conditions, static sinewave gratings were presented at 0.05 cycles/° for 500 ms at six orientations (0, 10, 45, 90, 100, 135°) and two contrasts (30, 100%), each followed by a 1 s blank screen at mean luminance. We presented gratings differing by only 10° in order to probe how small changes in orientation are encoded by the V1 population across luminance levels. Specifically, we tested whether the V1 population encoded this small orientation difference with equal fidelity across scotopic and photopic luminance levels. The results of this analysis have been presented previously, showing that the *d*′ for Δ10° has equal magnitude between scotopic and photopic luminance (O’Shea et al., 2025). For the analysis presented in Figures 2 and 7 in which we probed the V1 representation of mean luminance in response to otherwise identical stimuli, we compared the V1 population response to gratings at each of these six orientations and two contrasts, differing only in mean luminance. For the analyses in Figures 4 and 5, in which we ask how the V1 population encodes changes in orientation along with changes in luminance, only responses to pairs of gratings separated by 45° were considered. For the analysis in Figure 6*A*, in which we ask how the V1 population encodes changes in orientation along with changes in contrast, only responses to pairs of gratings separated by 45° were considered.

In the experiments presented in Figure 6*B*, drifting sinewave gratings were presented for 2,000 ms at eight orientations (0, 45, 90,135,180, 225, 270, 315°) and five spatial frequencies (0.02, 0.04, 0.08, 0.16, 0.32 cycles/°), each followed by a 1 s blank screen at mean luminance.

In the experiments presented in Figure 6*C*, drifting sinewave gratings were presented for 2,000 ms at one orientation (0°), five spatial frequencies (0.02, 0.04, 0.08, 0.16, 0.32 cycles/°), and four temporal frequencies (1, 2, 4, 8 Hz), each followed by a 1 s blank screen at mean luminance.

#### Light adaptation

At least 10 min prior to each experiment, the pupil was fully dilated with 1% atropine. Rod isomerization rates for each projector configuration were computed as previously described, using spectral radiance measurements taken with a PR-655 spectroradiometer (Rhim et al., 2021). In all experiments, stimuli were presented first under scotopic conditions and then under photopic conditions.

#### Scotopic adaptation

Scotopic luminance was achieved by lowering projector power and adding two 1% neutral density filters (Thorlabs) to the light path. Care was taken to black out any other sources of visible light during the experiment, and supervision of the experiment was done remotely in an adjacent room separated by a shut door and floor to ceiling black out curtains. Based on our spectral radiance measurements, this configuration generated 25 Rh* rod^−1^ s^−1^ [0.016 candelas (cd)/m^2^]. Previous work has established that this scotopic luminance level corresponds to the nighttime environmental luminance of a rural setting and drives solely rod-mediated responses in the retina (Spitschan et al., 2016; Rhim et al., 2021). Mice viewed a blank screen at mean luminance for 30 min prior to any stimuli being played to achieve scotopic adaptation.

#### Photopic adaptation

We then raised the projector power and removed the neutral density filters to generate photopic luminance at 1.13 × 10^5^ Rh* rod^−1^ s^−1^ (97 cd/m^2^). The photopic luminance level corresponds to midday sun and fully saturates rod-mediated responses in the mouse retina, with pupil dilated (Spitschan et al., 2016; Rhim et al., 2021; Franke et al., 2022). Mice viewed a blank screen at mean luminance for 10 min prior to any stimuli being played to achieve photopic adaptation.

### Quantification and statistical analysis

#### Imaging

All cellular fluorescence traces were baseline normalized on a single-trial basis with the mean fluorescence value in the 200 ms preceding stimulus onset. All visual responses were analyzed in units of percent change in fluorescence above baseline (Δ*F*/*F*):

$$\frac{\Delta F}{F}=\frac{F(t)-p}{p},$$

where *F*(*t*) is the raw fluorescence trace and *p* is the mean fluorescence magnitude in the baseline period.

For the luminance discrimination analysis, we compute each cell's response on a single-trial basis as the mean Δ*F*/*F* from 450 to 1,300 ms following stimulus onset, which captures the peak of the evoked fluorescence response to the 500 ms static gratings for cells expressing GCaMP6s.

To explore what sensory information is present in the high-dimensional V1 population code, we project the V1 population response across stimulus conditions into a two-dimensional space for computing the discriminability index (*d*′). We employ the “targeted dimensionality reduction” method for projecting neural population responses into an orthonormal basis which preserves the principal axis along which the population response shifts for a change in stimulus (dU) and the axis of the largest component of trial-to-trial covariance in the population response (*n*_1_; Heller and David, 2022).

For each orientation of the grating stimulus, we create two matrices of size *N*_cells_ × *N*_trials_, containing the single-trial responses for all simultaneously recorded cells at scotopic and photopic luminance. The difference between the trial-averaged means of these matrices gives the first axis in the decoding space—dU.

Next, we subtract the trial-averaged response for each stimulus condition from the response matrices and concatenate this zero-centered data into a “noise matrix” of size *N*_cells_  × 2*N_trials_. We then compute the first principal component of this noise matrix (*e*_1_), which represents the primary axis of trial-to-trial covariance in the V1 population response across stimulus conditions.

The axes dU and *e*_1_ are not necessarily orthogonal to one another. To form an orthonormal basis for dimensionality reduction, we compute the “noise axis” (*n*_1_) as the component of *e*_1_ which is orthogonal to dU. Neural data are then projected into the two-dimensional decoding space defined by the orthogonal axes dU and *n*_1_. The neural responses for each condition are now represented by a matrix of size 2 × *N*_trials_. In this two-dimensional space, we compute the difference in mean response to the two luminance conditions (Δ*U*) and the covariance across trials (Σ). The discriminability index (*d*′) is computed as follows:

$$d^{\prime }=\sqrt{\Delta U\;*\;\Sigma \;*\;\Delta U^{\prime }}.$$

To validate the generalizability of this decoding subspace for capturing the structure of the V1 population response, we hold out a random subset of response trials when fitting the space and compute *d*′ on the projection of this held-out data into the decoding subspace. Each grating stimulus was presented for 50 trials. To fit the decoding subspace, we select neural responses from a random set of 40 trials for each stimulus and use the remaining 10 trials to compute *d*′. We repeat this procedure for 50 iterations and compute the average *d*′ across all iterations, which we report as the *d*′ value in our results.

An analogous procedure was used to decode luminance from V1 population responses to natural scenes. We compute each cell’s response on a single-trial basis as the mean Δ*F*/*F* in 166 ms bins. Given the slow dynamics of the calcium indicator, this allows us to compare responses to every five frames of the natural movies. With these single-trial responses across luminance levels, we perform the same luminance decoding procedure as above.

#### Modeling

We simulate noisy V1 responses to repeated presentations of gratings at scotopic and photopic luminance via random draws from a multivariate normal distribution using the “mvnrnd” function in MATLAB. For the example simulations in Figure 5, the transition from scotopic to photopic visual stimulation is modeled by a doubling of the mean and variance of the multivariate normal distribution. This modeled V1 population response is then projected into the dDR space to compute *d*′ for luminance discrimination as described for the neural data in Figure 2.

#### Code accessibility

The MATLAB code to perform the analyses in this manuscript is available on Figshare (10.6084/m9.figshare.30359725). The imaging code is from the company Neurolabware and is freely available online (scanbox.org). The visual presentation code is available on GitHub (https://github.com/SNLC/ISI). We use the Suite2P postprocessing tools to extract cell masks and generate Δ*F*/*F* traces (https://github.com/MouseLand/suite2p).

## Results

**Figure 1.**
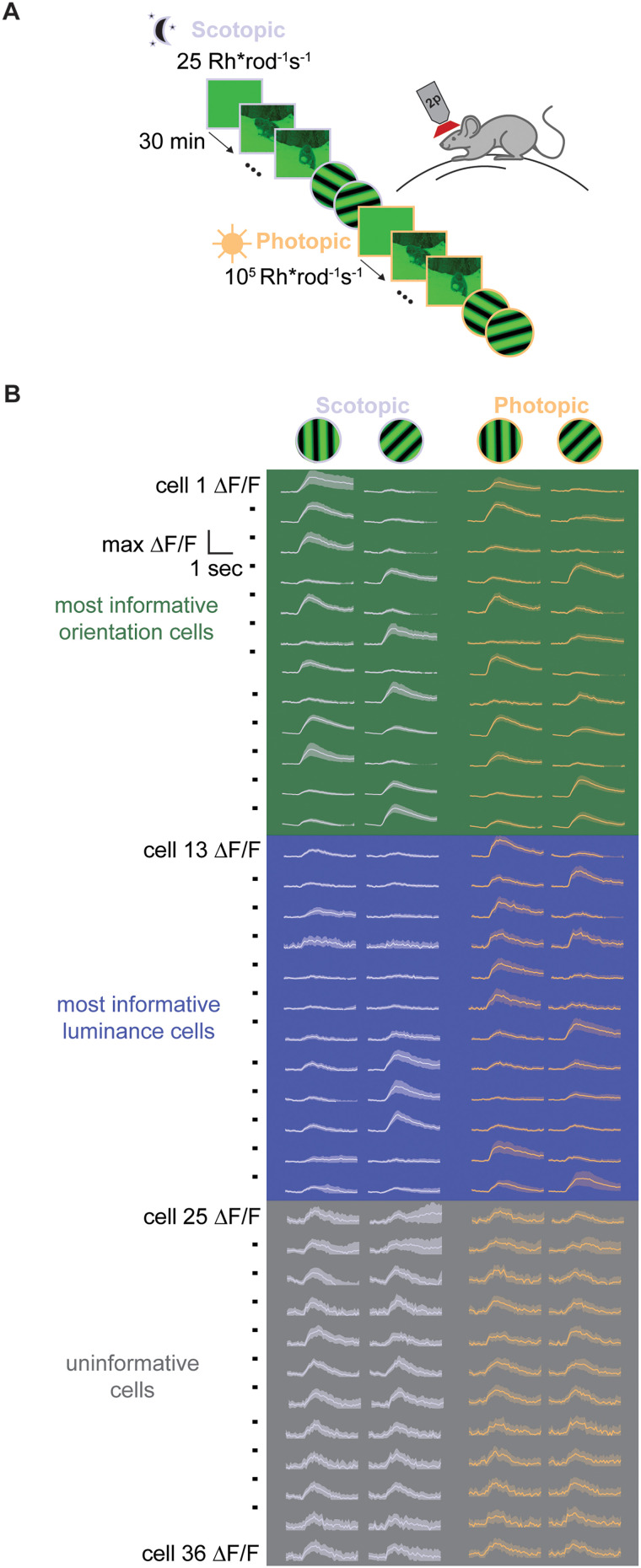
***A***, Experimental design. ***B***, Example fluorescent traces from simultaneously recorded V1 neurons responding to 0 and 45° gratings at scotopic and photopic luminance. For each cell, responses are scaled to the maximum Δ*F*/*F* across the four stimulus conditions. The solid line indicates the trial-averaged response, and the shaded region indicates ±1 standard deviation.

Mouse V1 represents spatiotemporal visual features, such as orientation, in a luminance-invariant manner (O’Shea et al., 2025). This luminance-invariant code could come about because information about luminance has been filtered out of the representation in V1. Alternatively, V1 could retain a representation of mean luminance level, but in a manner orthogonal to the representation of orientation. To determine whether the representation of mean luminance is retained in V1, we used two-photon calcium imaging to track the responses of V1 neurons to natural movies and oriented gratings across scotopic and photopic conditions (Fig. 1*A*).

We found a diversity of functional characteristics in the evoked responses of V1 neurons to pairs of oriented gratings differing by 45° across luminance conditions (Fig. 1*B*). Some cells encode this 45° orientation shift in a luminance-invariant manner (“most informative orientation cells”). A separate population of cells encodes a change in luminance by increasing response magnitude from scotopic to photopic states, while others have smaller evoked responses at higher luminance (“most informative luminance cells”). Still other cells exhibit evoked responses that are invariant to both changes in orientation and luminance and are therefore uninformative for representing these two stimulus variables (“uninformative cells”). These response motifs indicate that the V1 population encodes the mean luminance of visual scenes in tandem with an invariant representation of orientation and suggest that information about these two stimulus variables is carried by distinct neural populations, with responses modulated in a heterogeneous manner by changes in luminance.

**Figure 2.**
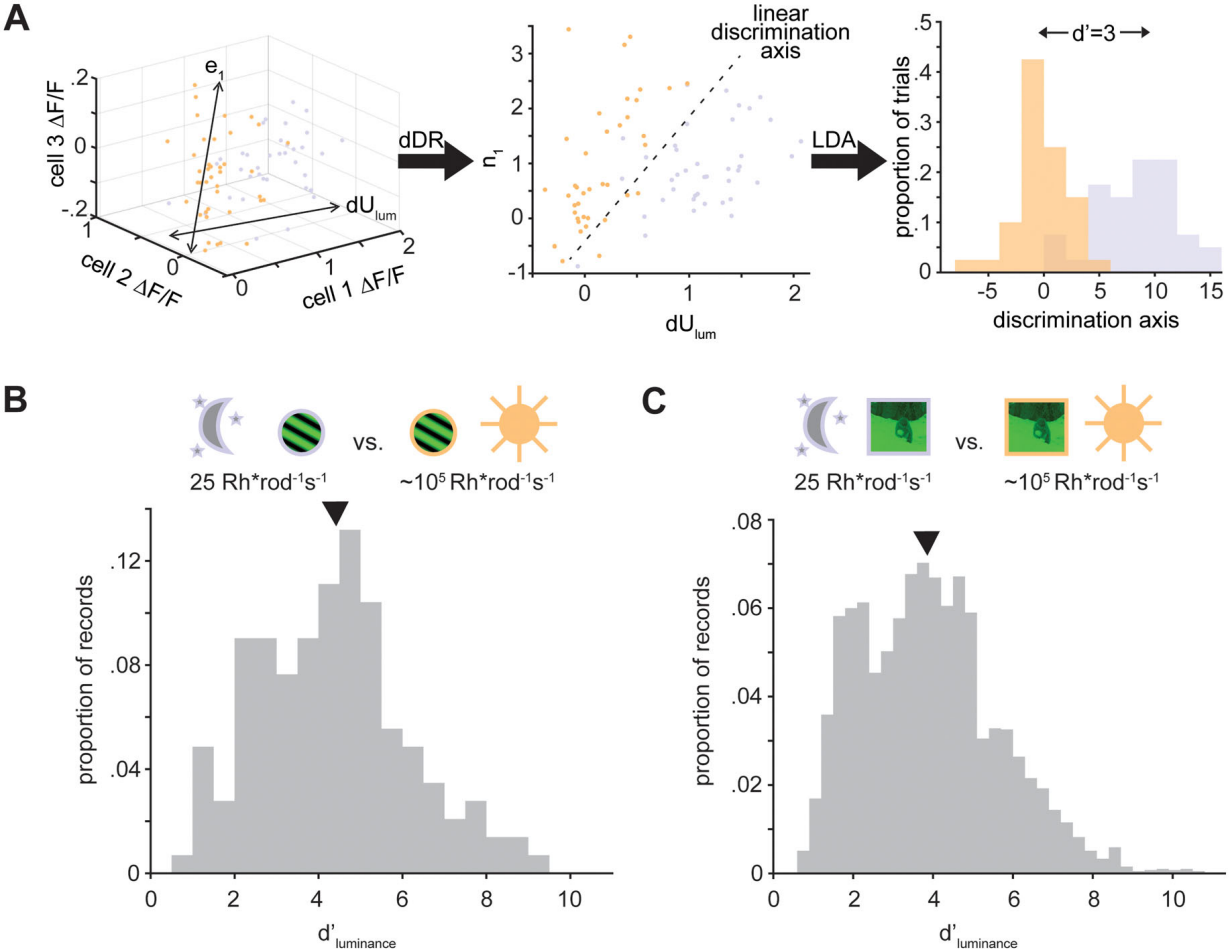
Discriminability of the V1 code for mean luminance. ***A***, Example of projecting neural responses into the dDR decoding space for luminance discrimination between photopic (orange) and scotopic (purple) conditions. ***B***, Histogram of *d*′ values across all V1 imaging experiments for discriminating responses to gratings of the same orientation at scotopic and photopic luminance. The black arrow indicates the mean across all experiments and grating orientations. ***C***, As in ***B***, for discriminating responses to natural movies at scotopic and photopic luminance.

To quantify the discriminability of the V1 population code for scotopic versus photopic luminance, we asked how well a linear decoding procedure could separate visually evoked population responses to the same orientation of gratings at scotopic (0.1 Rh*/rod/s) versus photopic (10^4^ Rh*/rod/s) luminance levels (*n* = 10 mice; 18 experimental sessions; 21,792 cells; 1,210.7 ± 740.9 cells/session). We projected neural population data into a two-dimensional decoding space using a dimensionality reduction method which captures the primary axes along which population responses varied across grating presentations (“dDR”; Fig. 2*A*; Heller and David, 2022). Luminance level was reliably encoded by the V1 population, indicated by large values of the discriminability index (*d*′; mean *d*′ = 4.33 ± 2.07; Fig. 2*B*). We performed the same luminance decoding procedure for V1 responses to natural scenes, and found that luminance level was also reliably encoded by the V1 responses to identical natural movies differing only in mean luminance (mean *d*′ = 4.18 ± 1.41; Fig. 2*C*).

10.1523/ENEURO.0281-25.2025.f2-1 Figure 2-1 Discrimination index for scotopic vs photopic luminance. A. Histogram of d’ values across all V1 imaging experiments for discriminating responses to gratings of the same orientation at scotopic and photopic luminance, plotted separately for 30% and 100% contrast gratings. Arrows indicate mean across all experiments and grating orientations at a single contrast. B. Scatter plot showing mean d'_luminance_ as a function of V1 population size. Black dots indicate mean across experimental stimuli pairs, errorbars indicate +/- .5 standard deviation. Download Figure 2-1, TIF file.

We investigated why the magnitude of *d*′ for luminance varied across experiments and stimuli (Fig. 2*B*,*C*). Some of the variability in *d*′ values across this dataset is due to diversity in both visual stimuli and experimental population size. *d*′ for discriminating luminance is significantly higher for gratings at 100% contrast (mean *d*′ = 5.1 ± 1.9) than at 30% contrast (mean *d*′ = 3.6 ± 1.8; *p* = 0.002, two-sample *t* test; Extended Data Fig. 2-1*A*). In addition, *d*′ for luminance discrimination scales with neural population size, in line with prior results showing a scaling in the fidelity of sensory encoding with neural population size (Pearson’s correlation = 0.68; Extended Data Fig. 2-1*B*; Kafashan et al., 2021; Stringer et al., 2021).

Having determined that the V1 population encodes luminance level, we asked how this representation coexists with the luminance-invariant representation of orientation by the mouse V1 population. Orientation can be decoded using a luminance-invariant projection of the V1 population response (O’Shea et al., 2025). Here we have shown that information on the luminance state can also be decoded from the same V1 population response. We compared our data to candidate models of V1 response distributions to explore how the V1 population jointly represents these two stimulus variables.

The population-level representation of orientation and luminance is built up from the signals carried by individual neurons. The change in mean response of each neuron for a change in stimulus orientation, dμ_ori_, or luminance, dμ_luminance_, quantifies how informative a cell’s trial-averaged response is for decoding the value of a particular stimulus variable. Cells with large changes in mean response magnitude for a change in stimulus value are the most informative for decoding (i.e., a “steep” tuning curve), while cells which have an invariant mean response across the range of stimulation are uninformative (i.e., a “flat” tuning curve). The vector of dμ values for all cells, dU, is the first axis of the decoding space and defines the principal dimension along which population activity varies with a change in the value of a stimulus variable of interest (Heller and David, 2022).

**Figure 3.**
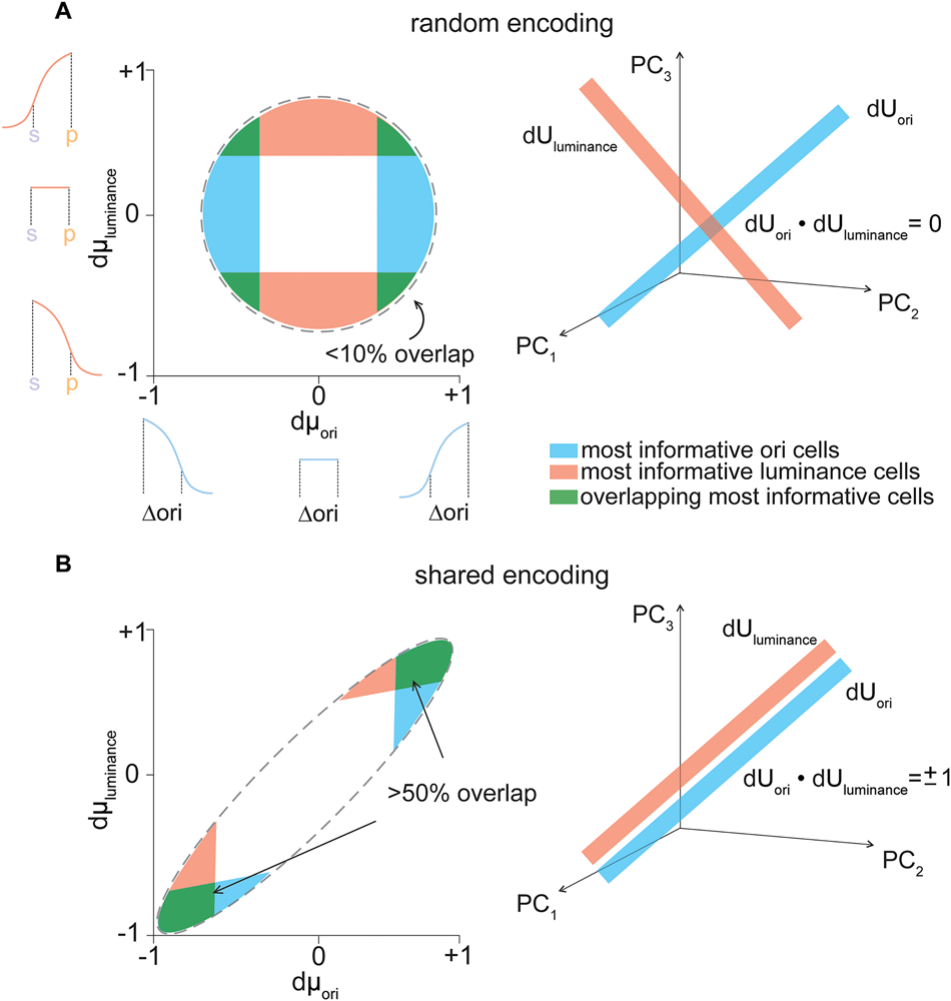
V1 population encoding models for the joint representation of orientation and luminance. ***A***, Illustration of the random encoding model. Left, Distribution of dμ_ori_ and dμ_luminance_ for model V1 cells. The most informative cells for decoding orientation and luminance are shaded in cyan and red, respectively. Overlap in the most informative subpopulations is shaded green. Inset tuning curves show the relative response magnitudes for changes in orientation and luminance corresponding to a large negative, zero, or large positive dμ values. Right, Three-dimensional representation of the modeled V1 population activity, showing the principal encoding axes for changes in orientation (cyan) and luminance (red). ***B***, As in ***A***, for the shared encoding model.

We considered two models for how luminance information is encoded by V1 cells in relation to their responses to changes in orientation. One possibility is that orientation and luminance information are distributed randomly across the V1 population, such that there is no correlation between the magnitude of dμ_ori_ and dμ_luminance_ for single cells (“random encoding model”; Fig. 3*A*, left). The correlation between the magnitude of dμ_ori_ and dμ_luminance_ determines the alignment of dU_ori_ and dU_luminance_, such that this encoding scheme generates orthogonal dU axes (dU_ori_·dU_luminance_ = 0) for orientation and luminance (Fig. 3*A*, right). Alternatively, dμ_ori_ and dμ_luminance_ could covary for a given cell, such that largely overlapping subpopulations of V1 cells are most informative for decoding both orientation and luminance (“shared encoding model”; Fig. 3*B*, left). In this encoding scheme, the dU axes are parallel for the two stimulus variables (dU_ori_·dU_luminance_ = ±1; Fig. 3*B*, right). Dot products between dU axes of both −1 and +1 correspond to the shared encoding model, as the sign of this axis alignment simply reflects the relative directions of the response shifts for given changes in orientation and luminance.

**Figure 4.**
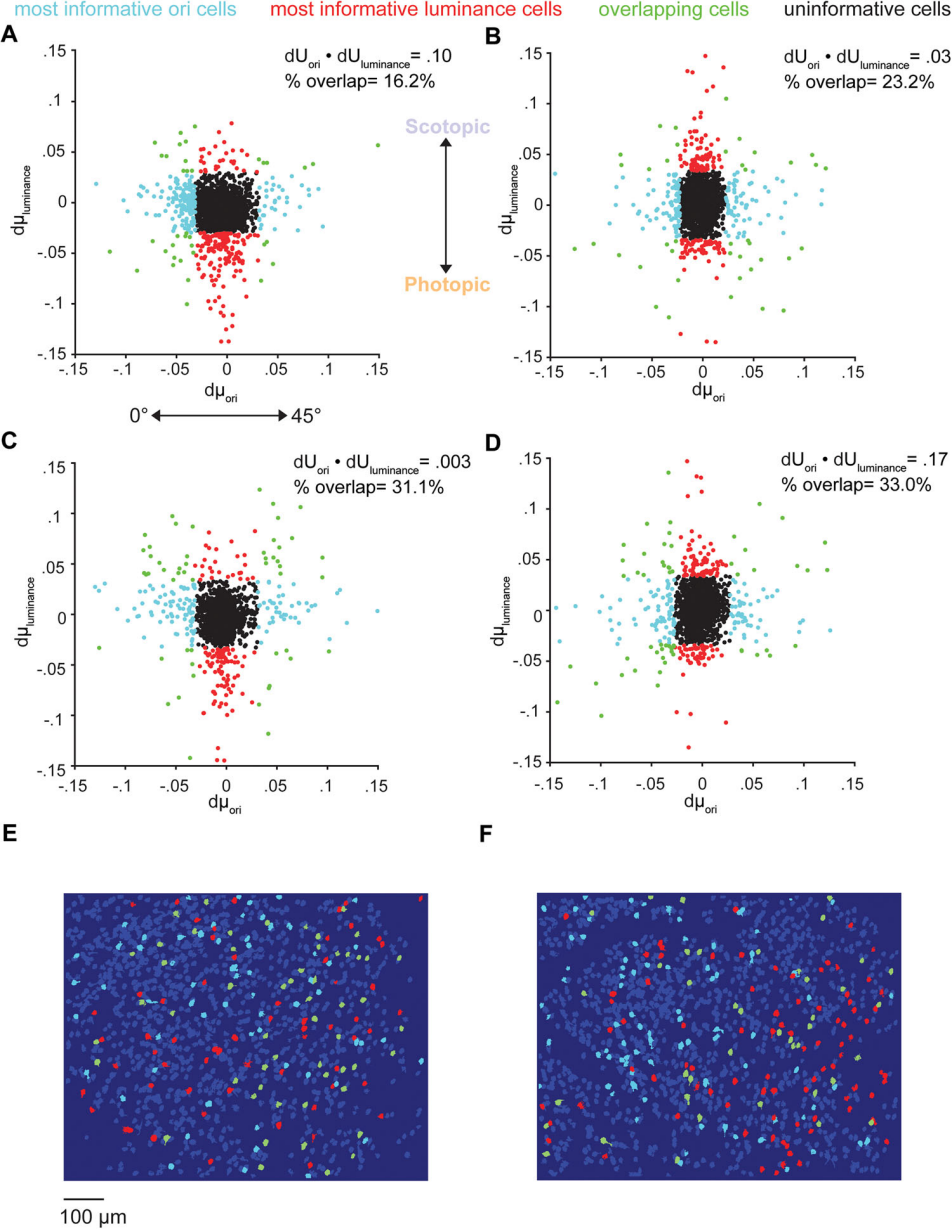
Experimental dμ_ori_ and dμ_luminance_ distributions. ***A–D***, Example V1 population dμ_ori_ and dμ_luminance_ distributions. Each scatter point gives the dμ_ori_ and dμ_luminance_ magnitude for a V1 cell. Top 10% largest dμ magnitude cells are highlighted for orientation (cyan) and luminance (red) discrimination. Cells that overlap in both most informative subpopulations are highlighted in green. ***E, F***, Example cell maps from V1 imaging experiments. The cells are highlighted according to the same color scheme as ***A***–***D***.

**Figure 5.**
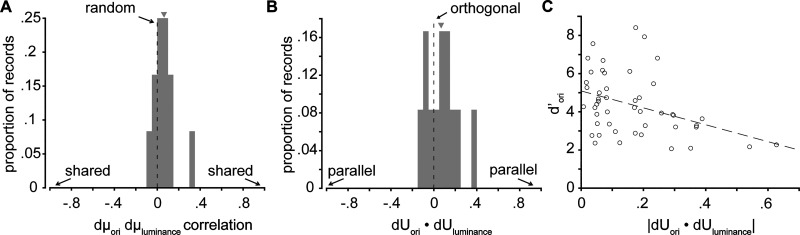
Joint representation of orientation and luminance by the V1 population response. ***A***, Histogram of the Pearson's correlation coefficient of dμ_ori_ and dμ_luminance_ for simultaneously recorded V1 cells. ***B***, Histogram of dU_ori_·dU*_l_*_uminance_ for each V1 population and stimulus set. ***C***, Scatter plot of the magnitude of dU_ori_·dU_luminance_ versus *d*′ for orientation discrimination. The arrows indicate means of distribution. All histograms show the average over stimulus orientation pairs for each experimental session.

To compare the V1 population data to these candidate encoding schemes, we computed the change in trial-averaged response of all V1 cells to 45° changes in the orientation of 100% contrast, static gratings (dμ_ori_) and shifts from scotopic to photopic luminance (dμ_luminance_; *n* = 8 mice; 12 experimental sessions; 14,320 cells; 1,193.3 ± 734.4 cells/session). dμ_ori_ and dμ_luminance_ were on average uncorrelated when plotted against each other for all cells in each experimental population (mean Pearson's correlation coefficient = 0.07 ± 0.14; Figs. 4*A*–*D*, 5*A*). These scatter plots reveal additional, important characteristics of the joint encoding of orientation and luminance by the V1 population. First, the distribution of dμ_ori_ is symmetric about zero, indicating that equal numbers of V1 neurons increase as they decrease their response for a particular 45° change in orientation. In addition, an increase in luminace from scotopic to photopic levels causes bidirectional shifts in response magnitude across the V1 population; some neurons increase their responses, while other neurons decrease their responses. On average, however, V1 cells show an increase in response magnitude for photopic versus scotopic luminance, as shown previously (mean log ratio = 0.33 ± 0.79; O’Shea et al., 2025). This differs from the joint encoding of orientation and contrast, where an increase in contrast is encoded by a unidirectional increase in response magnitude across the V1 population (mean log ratio = 0.83 ± 0.75; Extended Data Fig. 6-1*A*).

In line with the response motifs observed across the V1 responses from Figure 1, these scatter plots suggest that information about changes in orientation and luminance is carried along independent dimensions of the V1 population response. As predicted by the random encoding model, there was some overlap between those neurons that are most informative about luminance and orientation (Fig. 3). We defined the “most informative” neurons for orientation or luminance discrimination as those with the top 10% dμ magnitude or the largest trial-averaged change in response as a function of a change in stimulus. On average, there was 34 ± 07% overlap in the “most informative” neurons for the two discrimination tasks. These functionally distinct subpopulations are spatially intermingled across the V1 imaging plane (Fig. 4*E*,*F*).

Since dμ_ori_ and dμ_luminance_ were uncorrelated for individual neurons, dU_ori_ and dU_luminance_, the primary axes for decoding stimulus information from the population response, tended to be orthogonal (mean dot product = 0.08 ± 0.20), consistent with the prediction of the random encoding model (Fig. 5*B*). While we observed some variability in the alignment of dU_ori_ and dU*_l_*_uminance_ across stimulus pairs, there was a clear correlation between *d*′ for orientation discrimination and the orthogonality of dU_ori_ and dU_luminance_ across experiments and stimulus pairs, such that V1 populations with the highest fidelity population code for orientation were those which encoded orientation and luminance along orthogonal dimensions in the neural response space (Pearson's correlation coefficient = −0.39; Fig. 5*C*). This analysis indicates that, in keeping with the predications of the random encoding model, the V1 population encodes orientation and luminance independently, resulting in orthogonal decoding axes for these two stimulus variables.

Next, we asked how the V1 population jointly encodes other visual stimulus properties. In particular, does the V1 population encode additional stimulus variables along orthogonal axes, as for the representation of orientation and luminance, or do these representations covary?

**Figure 6.**
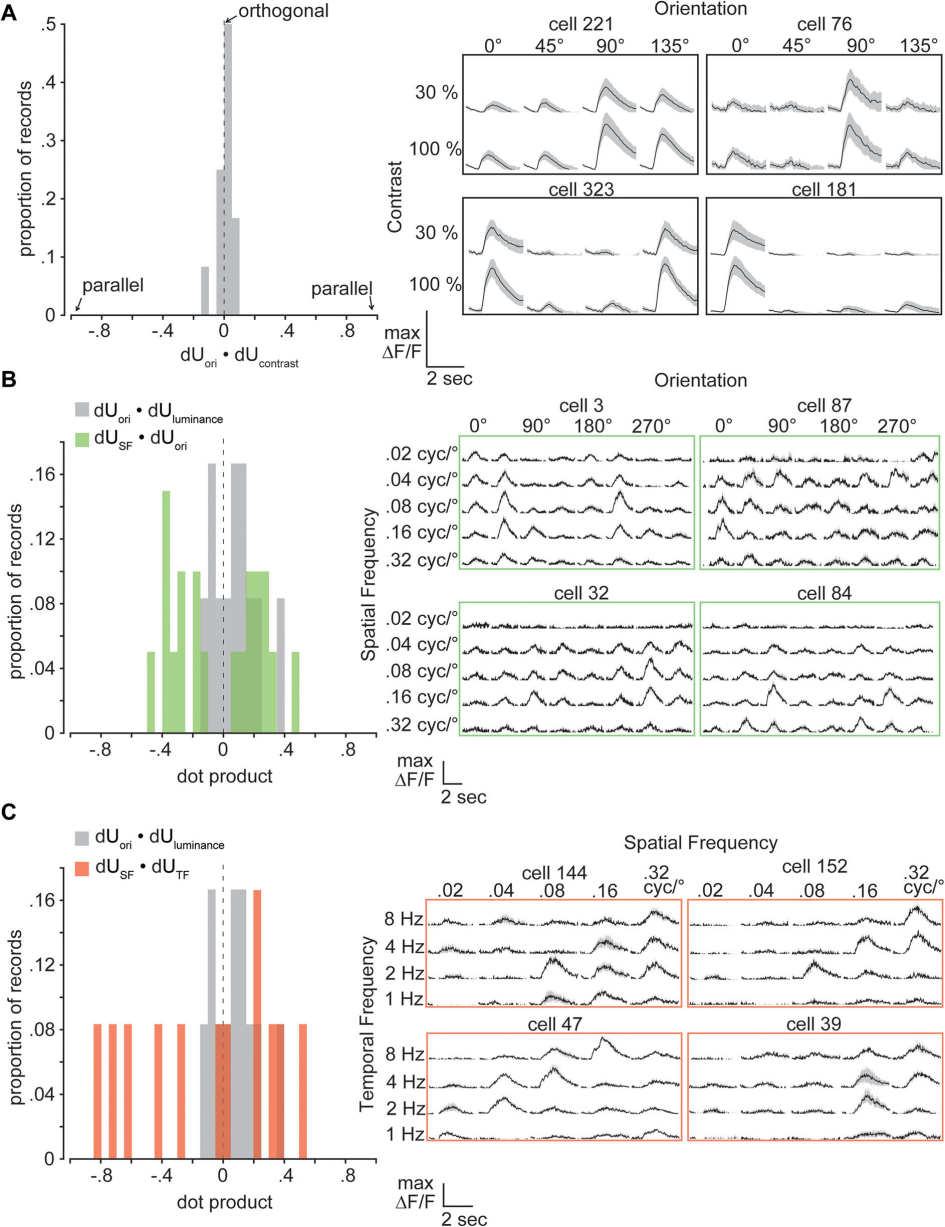
Joint representation of visual stimulus features by the V1 population response. ***A***, Left, Histogram of dU_ori_ · dU_contrast_ (right) example fluorescent traces from V1 cells responding to static gratings across orientations and contrasts. The solid line indicates the trial-averaged response, shaded region indicates ±1 standard deviation. ***B***, As in ***A***, for orientation on SF. ***C***, As in ***A***, for SF and TF. All histograms show the average over stimulus orientation pairs for each experimental session.

Given that V1 neurons exhibit contrast-independent orientation tuning, we predicted that orientation and contrast would be represented independently by the V1 population response (Fig. 6*A*; Sclar and Freeman, 1982). Scatter plots for dμ_ori_ versus dμ_contrast_ indicate that orientation and contrast information are carried by V1 subpopulations matching a random encoding model (Extended Data Fig. 6-1*A*). In keeping with this result, we find that dU_contrast_ and dU_ori_ tend to be orthogonal, which represents a generalization of contrast-invariant orientation tuning at the single-cell level to the mouse V1 population code (mean dot product = 0.01 ± 0.29; Fig. 6*A*). Importantly, unlike the representation of luminance in V1, changing contrast has a unidirectional effect on V1 response magnitudes (Extended Data Fig. 6-1*A*).

10.1523/ENEURO.0281-25.2025.f6-1 Figure 6-1 Experimental dμ and dμ distributions. A. Example V1 population dμ_ori_ and dμ_contrast_ distributions. Each scatter point gives the dμ_ori_ and dμ_contrast_ magnitude for a V1 cell. Top 10% largest dμ magnitude cells are highlighted for orientation (cyan) and contrast (red) discrimination. Cells that overlap in both most informative subpopulations are highlighted in green. B. As in A, for dμ_ori_ and dμ_SF_. C. As in A, for dμ_SF_ and dμ_TF_. Download Figure 6-1, TIF file.

We found that not all visual stimulus features are encoded along orthogonal dimensions by the V1 population. The orientation tuning of mouse V1 neurons varies with spatial frequency (SF), suggesting that orientation and SF are not encoded along orthogonal dimensions in the neural response space (Fig. 6*B*; Ayzenshtat et al., 2016; Pattadkal et al., 2018). To test this idea, we computed decoding axes for V1 population responses to 45° changes in the orientation of drifting gratings and to 1 octave changes in the SF of gratings at a fixed orientation. Example scatter plots for dμ_ori_ versus dμ_SF_ are presented in Extended Data Fig. 6-1*B*. dU_ori_ and dU_SF_ were not orthogonal, but rather had dot products shifted away from zero, in contrast with the orthogonality of dU_ori_ and dU_luminance_ (Fig. 6*B*).

Similarly, we computed the alignment of decoding axes for 1 octave changes in the SF and temporal frequency (TF) of gratings, since mouse V1 neurons do not encode these variables independently but rather exhibit tuning for SF–TF combinations corresponding to a particular velocity (Fig. 6*C*; Priebe et al., 2003; Andermann et al., 2011). Example scatter plots for dμ_SF_ versus dμ_TF_ are presented in Extended Data Figure 6-1*C*. dU_SF_ and dU_TF_ were also not orthogonal (Fig. 6*C*).

The representation of luminance by the V1 population could be due to a change in the gain of visually evoked responses in the retina between scotopic and photopic conditions. RGC firing rates are higher in the photopic than scotopic regime, and this difference could be inherited by V1 in the form of a one-dimensional gain shift of evoked responses across the population (Ruda et al., 2020). This kind of change in response magnitude across the V1 population is one way in which V1 might encode the luminance level. We simulate this scenario by taking a set of V1 responses to two gratings in the scotopic regime and multiplying them by a factor of two to get a pseudo population response for the photopic condition (Extended Data Fig. 7-1*A*, left panel). This pseudo population response carries information about both the orientation and luminance of the stimulus. In this scenario, equalizing response magnitudes across the two luminance conditions, by normalizing the population response to the maximum response within each condition, eliminates the luminance information in the V1 code, without disrupting the representation of orientation (Extended Data Fig. 7-1*A*, right panel).

**Figure 7.**
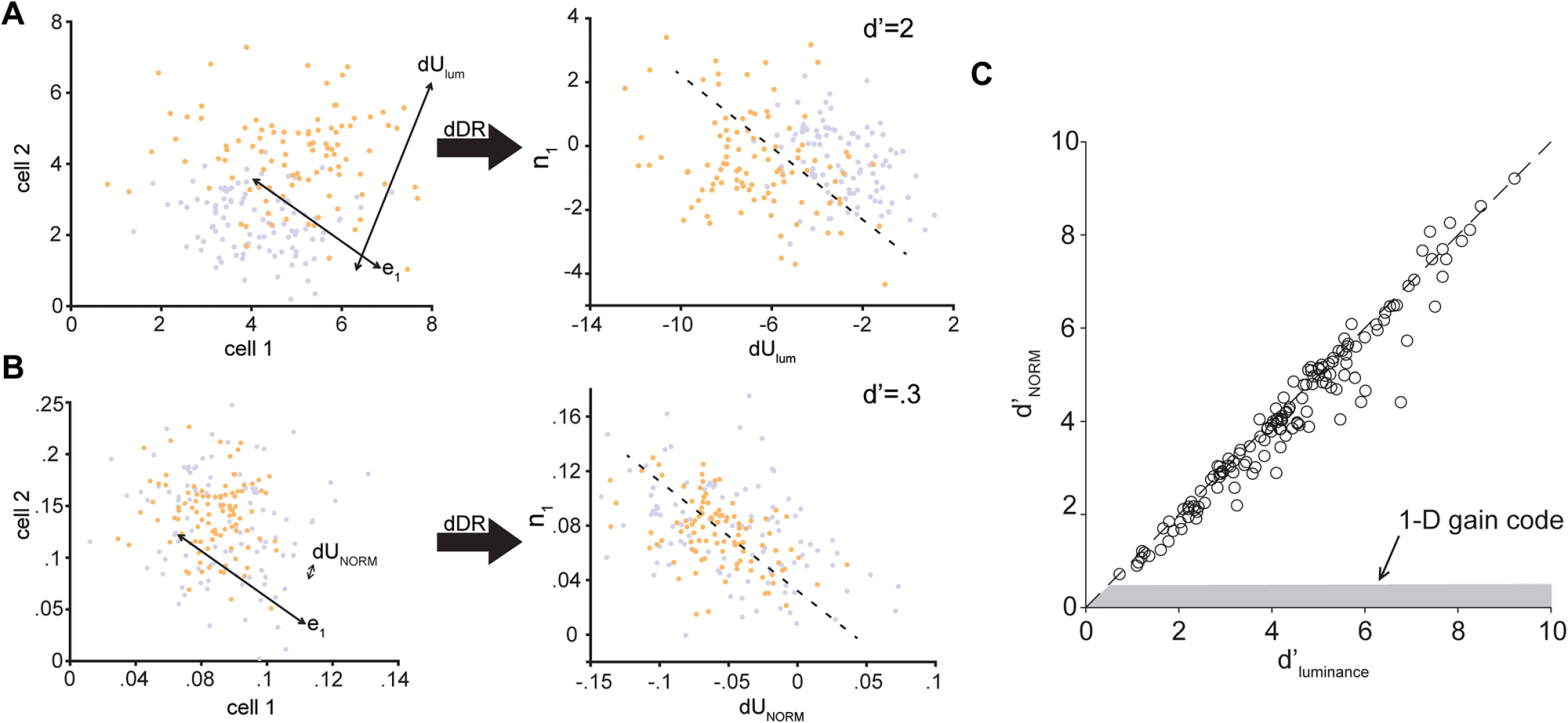
Luminance information is not carried by a scalar gain change in the V1 population response. ***A***, Schematic of a two-neuron model for luminance encoded by a scalar gain change in responses, which increases the mean and variance of the neural responses by a factor of 2 in the photopic (orange) relative to scotopic (purple) conditions. Right, Luminance condition can be discriminated from these population responses using the dDR method. ***B***, As in ***A***, following normalization of responses. ***C***, Scatter plot showing *d*′ for luminance in experimental V1 data before and after normalizing response magnitude in each condition. The gray region shows the prediction of the effect of normalization on luminance discriminability for a one-dimensional gain change in the V1 population response with luminance.

To further illustrate how luminance could be encoded by a change in the gain of V1 responses, consider a model two-neuron population responding to the same stimulus at two different luminance levels, with the only change in the responses across conditions being a scalar shift in the mean and variance, akin to V1 neurons inheriting a gain shift from the retina across light levels (Fig. 7*A*, left panel). A linear decoder can separate responses from the two conditions with high fidelity (Fig. 7*A*, right panel). However, if we normalize the modeled responses to the maximum response within each condition, which eliminates the change in response magnitude between luminance conditions, then the two conditions are no longer linearly discriminable (Fig. 7*B*). This modeling result generalizes to networks of arbitrary size.

10.1523/ENEURO.0281-25.2025.f7-1 Figure 7-1 Modeling V1 responses to scotopic and photopic luminance. A. (left) Modeled fluorescent traces from V1 neurons responding 0° and 45° gratings at scotopic and photopic luminance. The photopic responses are the scotopic responses scaled by a factor of 2. (right) Modeled fluorescent traces from V1 neurons following normalization to the maximum response across all neurons in each light condition. B. (left) Real fluorescent traces from simultaneously recorded V1 neurons responding 0° and 45° gratings at scotopic and photopic luminance. (right) Real fluorescent traces from V1 neurons following normalization to the maximum response across all neurons in each light condition. Solid line indicates the trial-averaged response, shaded region indicates +/- 1 standard deviation. Download Figure 7-1, TIF file.

In order to determine whether the representation of luminance by the mouse V1 population is due to a scalar shift in response magnitude across the population, we applied the same normalization procedure to our real neural data. Normalizing the V1 population response within each condition does not disrupt that representation of luminance (Extended Data Fig. 7-1*B*). This is because the transition from scotopic to photopic luminance has heterogeneous effects on responses across cells. These changes are distributed across the V1 population and are not one-dimensional, like a shift in gain. Rather, the effect of changing luminance is high-dimensional, and therefore not eliminated through this normalization procedure. We found that *d*′ values for luminance discrimination are unaffected by this normalization, indicating that the representation of luminance level in V1 is not solely due to a one-dimensional gain shift fed forward from the retina (mean *d*′ ratio = 0.95 ± 0.09; Fig. 7*C*).

## Discussion

We have shown that the mouse V1 population encodes mean luminance and orientation in a separable manner. Information about the orientation and luminance of visual inputs is distributed randomly across the V1 population, such that the principal decoding axes for these two stimulus variables are orthogonal to one another. These results indicate that the V1 population response carries information about mean luminance, in a manner that does not interfere with using a shared, linear decoder to recover orientation information across scotopic and photopic conditions. Thus, V1 encodes visual orientation and mean luminance independently, allowing for a luminance-invariant spatial representation of visual features to coexist with a flexible representation of environmental state. The joint representation of orientation and luminance is distinct from that of orientation and contrast, which are also encoded along orthogonal axes by the V1 population, in that a change in luminance has diverse, bidirectional effects on response magnitudes across the V1 population. The encoding of orientation and luminance is also distinct from the encoding of other visual properties, such as spatial and temporal frequency, which are not encoded independently by the V1 population.

The visual system must encode not only spatiotemporal information related to the identity and motion of objects in visual scenes but also information about the current state of the environment. The mean luminance of a visual scene is a valuable measure of environmental state as it systematically shifts on a daily basis. Importantly, most RGCs do not encode absolute luminance level in a monotonic manner. Rather, luminance adaptation shifts the gain of RGC responses to retain sensitivity over a relatively narrow range of light intensities.

How then might downstream visual circuits extract information about the mean luminance of a visual scene? One possibility is that the luminance adaptation state is encoded implicitly via functional shifts in the spatiotemporal or chromatic responses of neurons in central visual areas. Functional shifts which are reliably tied to a change in mean luminance provide information about the mean luminance of the visual input. For example, species with a rod-free fovea, such as primates, lose visual sensitivity at the center of the visual field when adapted to luminance below the threshold for cone activation. This functional shift in the output of the retina alters the visual information available to downstream circuitry. In primates, a simple representation of the luminance adaptation state could be extracted from whether or not V1 neurons with receptive fields within the foveal retinotopy are visually driven. Importantly, outside of the foveal zone, the spatial selectivity of macaque V1 cells is largely invariant across scotopic and photopic conditions, as in the mouse (Duffy and Hubel, 2007; O’Shea et al., 2025). Furthermore, human psychophysical experiments have shown that the perceptual attenuation of spatial and temporal contrast sensitivity for low frequencies, first measured under photopic conditions in human subjects, persists in scotopic conditions. In addition, the perceptual phenomenon of simultaneous contrast, in which squares of equal luminance appear to differ in brightness depending on the luminance of the surrounding area, persists in the scotopic regime (Fiorentini and Maffei, 1973). These results indicate that luminance-invariant spatial encoding is a general property of mammalian V1 and raise the question of how luminance could be represented by V1 neurons in the absence of changes in receptive field structure.

Our results, along with prior psychophysical experiments, indicate that changes in mean luminance are encoded by V1 despite luminance adaptation in the retina and the luminance-invariant tuning properties of downstream neurons. These results suggest that an explicit signal for luminance, perhaps originating in the ipRGCs, provides a representation of luminance to downstream visual areas. Indeed, ipRGCs project to the lateral geniculate nucleus of the thalamus (LGN), implicating this class of neurons in the thalamocortical visual circuit (Dacey et al., 2005; Brown et al., 2010). ipRGCs have overlapping spectral sensitivity with mouse rod and M-cone photoreceptors and will therefore be differentially activated by the 525 nm scotopic and photopic stimuli used in this study. Humans and mice can behaviorally discriminate the brightness of visual metamers that do not alter rod or cone activation, but do differentially activate melanopsin, the photopigment present in ipRGCs (Brown et al., 2012). Finally, mice lacking rod and cone photoreceptors can still perform a visually guided task in which they must discriminate the brightness of two displays in order to exit a water maze (Brown et al., 2012). These findings suggest that signals from ipRGCs projecting to the LGN could modulate the V1 population response across light levels.

Here we have shown that the V1 representation of mean luminance is not due to a one-dimensional shift in the gain of the population response. Rather, changing luminance has diverse effects on individual V1 neurons, with some neurons showing no change in visually evoked response magnitudes between luminance conditions, others having higher response magnitudes for scotopic relative to photopic stimuli, and still others responding more strongly in the photopic than scotopic condition. The finding that the effect of transitioning from scotopic to photopic luminance on V1 activity is high-dimensional emphasizes the importance of studying visual encoding from a neural population perspective, since the representation of luminance is distributed across V1 neurons which respond to changes in luminance in a heterogeneous manner.

Our observation that luminance can modulate V1 neuronal responses bidirectionally and with varying magnitudes may be at odds with the canonical finding that ipRGCs respond monotonically to increases in luminance (Do and Yau, 2010). Any proposed circuit mechanism for the encoding of luminance in V1 must account for this bidirectional modulation of V1 responses across the population, given the monotonic modulation of ipRGC firing rate by luminance. Interestingly, recent studies of ipRGCs in the mouse retina have identified functional clusters of ipRGCs which have distinct dynamic ranges for encoding luminance (Sondereker et al., 2020). Some classes of ipRGCs are more strongly driven at scotopic than photopic luminance, breaking from the canonical view of ipRGCs as monotonic encoders of luminance. A possible explanation for the diverse effects of luminance on V1 responses could be that projections from these functional clusters of ipRGCs remain segregated in downstream visual areas.

The visual system must convert retinal activity into a code for guiding behavior. Constructing such a code requires that the representations of certain visual features be independent of one another. For example, objects must be identified regardless of the environmental conditions in which they appear. Here we have shown that a representation of absolute luminance coexists with an invariant representation of spatial visual information in mouse V1. Further, we demonstrate how visual representations are distributed across the cortical population. By following a random coding scheme, in which there is no correlation between a neuron's change in response to a change in stimulus orientation and the change in response to a change in luminance, these two stimulus variables can be encoded independently by the V1 population.

The joint encoding of visual features by a random encoding scheme, as we have described for orientation, luminance, and contrast, is one way in which the visual system extracts behaviorally relevant information from retinal signals. There are other mechanisms for generating independent representations, such as encoding features by separate neural populations, either within or across brain regions. While V1 generates separable representations for orientation and luminance, the linked representations of SF and TF could indicate either that a separable representation for these features has not yet been unraveled at this stage in the visual hierarchy, or, more likely, that another representation is more important for driving behavior: sensitivity to speed. Understanding which visual feature representations are linked and which are encoded independently by neural populations across visual areas will reveal the process by which the visual system extracts behaviorally relevant information from retinal signals.

## Synthesis

Reviewing Editor: Fabienne Poulain, University of South Carolina Decisions are customarily a result of the Reviewing Editor and the peer reviewers coming together and discussing their recommendations until a consensus is reached. When revisions are invited, a fact-based synthesis statement explaining their decision and outlining what is needed to prepare a revision will be listed below. The following reviewer(s) agreed to reveal their identity: Jason Triplett, Bryan Hooks.

In this manuscript, the authors explore the intriguing question of how primary visual cortex (V1) encodes luminance information in relation to orientation of stimulus. They used Ca2+ imaging to monitor the activity of large ensembles of neurons that are presented multiple types of stimuli under scotopic and photopic conditions. To analyze these data, the authors developed a novel quantitative index to evaluate the ability of individual neurons to discriminate orientation and luminance. They make the novel observation that luminance and orientation are likely encoded by distinct neuronal populations in V1, which is in accordance with a model of random, as opposed to shared, encoding. They also present evidence that luminance encoding in V1 is not mediated by an inheritance of gain form the retina. Although some data seem to largely overlap with an earlier publication ("Luminance invariant encoding in mouse primary visual cortex" O'Shea et al., Cell Reports 2025), the finding that luminance level is encoded and does not vary with stimulus orientation (called orthogonal encoding) is new. Overall, the findings presented in that manuscript will be of interest to the field, but several major concerns about the presentation of the data and specifics on the methods used to determine tuning need to be addressed to solidify and strengthen the manuscript.

Major Concerns:

1) Almost all the figures present results of analysis while showing very little or none of the original experiments or imaging. Thus, it is difficult as a reviewer to assess the original data and whether the results of the analysis hold up. It would be nice to see some of the raw data in the paper (e.g. what would the mean Ca2+ signals from 1000+ cells look like when plotted for different luminance levels? While the decoder can determine what the luminance level is, is it also obvious to the human eye and would we be able to detect what the pattern is?)

2) The methodology to derive discriminability index is a bit opaque. To make the findings more accessible to a wider audience, a more detailed explanation of the rationale behind the quantitative methods, as well as a clearer description of how the dU and e1 components were computed is needed. For instance, the determination of dU (first axis) makes pretty good sense - the difference in response across all trials in different luminance conditions. However, the second axis is hard to decipher. The first principal component of all zero-centered (z-scoresd?) responses is determined (e1), but "The second axis in the decoding space is the component of e1 which is orthogonal to dU- the noise axis." It is unclear what this means nor why the first principal component was not used. Furthermore, the equation for d' is shown and dU is defined but not dU' (is this the orthogonal?), and E is defined as "the 2x2 covariance matrix." Which covariance matrix?

3) Fig. 1 shows a schematic of the recording paradigm and a schematic of how d' was determined from dU and e1, however, the axes in the second and third plot in Fig. 1B are not labeled and the transformation performed between each is unclear. To the point above including a schematic of how raw responses are translated into dU and e1 would be helpful, as well. Since Fig. 1 jumps straight to a population distribution of the discriminability index, there are no representative examples nor averaged responses of actual neuronal data. It would help immensely to show what type of response variability was observed on at least a few representative neurons. i.e. ones that changed firing in response to luminance but not orientation and vice-versa.

4) Each of the distributions in Fig. 1C and D seem to have two (or more peaks). Are these meaningful (i.e. different populations?). The focus here is on discriminability for luminance, but what about the distribution of neurons based on orientation discriminability? Or a more simple measure such as orientation selectivity?

5) In Fig. 5, the authors present an argument that luminance encoding in V1 is not mediated by inheriting gain from the retina. Again, presentation of actual responses of luminance informative cells vs. overlapping vs. orientation informative cells would be useful here. In addition, discussion (or even better, exploration) of an alternative model for luminance encoding by V1 would substantially strengthen the manuscript.

Minor Concerns:

1) Figure 1: The font size is so small in Fig. 1B as to be nearly illegible. Thus, in the dDR plot for decoding orientation nor in the LDA (? Hard to read) plot (natural scenes) can we really see what the x- and y-labels are.

2) Figure 3: This plot supports the finding that V1 cells with a strong encoding for orientation were those which encoded orientation and luminance along orthogonal dimensions in the neural response space. However, the color scheme in Fig. 3 makes it hard to discern which cells belong to which group. The plot might be more easily interpretable by indicating there are blue dots for orientation selective only cells (near 0 in du-luminance) and pink dots for luminance selective only cells (near 0 in du-ori), and these are in difference places. However, the proximity of large numbers of black dots (presumably not informative for either) and some number of gray dots at the periphery of the black cloud) make it harder to tell what's going on in the figure. There seems also to be some green (both) data, but the color contrast is not easy for me to tell. Are there gray dots or is this overlap of other categories, could the legend explicitly state the black is nonselective?

3) In the description of the decoding calculation, line 464-465 mentions "held-out" trials projected into decoding space. Which trials are these? Why were all data not included?

4) The rationale behind the choices of orientations shown is not described. For drifting gratings a typical 45deg spacing is used, but for static gratings a somewhat random array is used. If this is so comparisons between 10deg and 45deg differences can be compared, this is not explicitly stated anywhere. The only mention present was analysis of 45deg changes in calculating orientation (line 177). Why not use something more informative like an orientaton-selectivity index?

5) How does this vary across species? It would be nice to indicate if this has been tested in other animals (e.g. how do I as a human know luminance level? I feel when I read indoors and outdoors that the page looks largely the same, even though (a) I know the photon flux is much much (several orders of magnitude) lower indoors, and (b) Even though is most looks the same ... there is some awareness that it's brighter or dimmer in the environment ... but no idea how our brains do that. Is there some proposed circuit mechanism? Or a simplified idea of how the detector can tell what the luminance level is, and does this vary across humans/mice?

6) At very low luminance (I'm gathering lower than attempted here), the ability to detect anything at all must go to chance, so is there some threshold level which must be reached to have luminance invariance of orientation selectivity?

7) Line 200+: "To test this idea, we computed decoding axes for V1 population responses to 45{degree sign} changes in the orientation of drifting gratings, and to 1 octave changes in the SF of gratings at a fixed orientation." Could this data be plotted as in Fig 3.? It seems weird to not show how the results look different when plotted for a different pair of parameters to decode but instead plot more derived features in Fig. 4). This also applies to the temporal frequency.

8) Line 218+: If the "most informative" neurons for orientation or luminance discrimination are "those with the top 10% dm magnitude, or the largest trial-averaged change in response as a function of a change in stimulus", then why isn't the line separating the red/blue/black data in Fig. 3 more straight? Or maybe it is straight and the gray dots make it hard to tell?

9) Figure 5 explores whether a linear decoder can separate responses from two luminance conditions assuming the effect on firing is simply a change in gain. This seems to be performed with modeled data? Perhaps I misunderstand (Line 255: "if we normalize the modeled responses").

10) Line 279: "We have shown that the mouse V1 population encodes mean luminance and orientation in a separable manner." Can you give some insight into the circuit or synaptic mechanism? Do we understand why these are separable? Is it just the separate cell populations encode each (e.g., let's take the orientation selective ones as one population, and luminance selective as separate groups that just happen to be spatially intermingled)? Are these just a random subset of the whole neuron population? (e.g. we could do it with 10% for luminance and 10% for orientation and the other 80% are set aside for ... other orientations or characteristics?) Are they spatially intermingled in other mammals (cat, primate)?

11) How might downstream visual circuits extract mean luminance? One suggestion is a "functional shift" in the "spatiotemporal or chromatic responses of neurons in central visual areas". Wouldn't cat/human be better to study this question since the central-to-peripheral gradient is somewhat steeper in the retina of these animals?

12) ~Line 314: The result "We found, however, that normalizing V1 response magnitude across light levels did not alter the ability to decode luminance state suggesting that additional signals are present in V1 that are related to luminance state" is somewhat not fully satisfying since it suggests that firing rate is not needed to decode luminance but leaves unanswered perhaps what does suffice? The ipRGC hypothesis is interesting but could be tested.

## Author Response

Synthesis of Reviews:

Computational Neuroscience Model Code Accessibility Comments for Author (Required):

The authors have not made the custom MATLAB codes they used for imaging, stimulus presentation and modeling available.

We now have added a section to our methods called "Code Accessibility" which describes how to access the software employed in this study. We have placed the MATLAB code to perform the analyses we present in Figshare (10.6084/m9.figshare.30359725). The imaging code is from the company Neurolabware and is freely available online (scanbox.org). The visual presentation code is available on github (https://github.com/SNLC/ISI). We use the Suite2P postprocessing tools to extract cell masks and generate dF/F traces (https://github.com/MouseLand/suite2p).

Synthesis Statement for Author (Required):

In this manuscript, the authors explore the intriguing question of how primary visual cortex (V1) encodes luminance information in relation to orientation of stimulus. They used Ca2+ imaging to monitor the activity of large ensembles of neurons that are presented multiple types of stimuli under scotopic and photopic conditions. To analyze these data, the authors developed a novel quantitative index to evaluate the ability of individual neurons to discriminate orientation and luminance. They make the novel observation that luminance and orientation are likely encoded by distinct neuronal populations in V1, which is in accordance with a model of random, as opposed to shared, encoding. They also present evidence that luminance encoding in V1 is not mediated by an inheritance of gain form the retina. Although some data seem to largely overlap with an earlier publication ("Luminance invariant encoding in mouse primary visual cortex" O'Shea et al., Cell Reports 2025), the finding that luminance level is encoded and does not vary with stimulus orientation (called orthogonal encoding) is new. Overall, the findings presented in that manuscript will be of interest to the field, but several major concerns about the presentation of the data and specifics on the methods used to determine tuning need to be addressed to solidify and strengthen the manuscript.

A major issue raised by the reviewers was that we did not present examples of raw data from our imaging experiments to illustrate our findings. We have corrected this oversight throughout the manuscript by presenting examples of V1 responses which illustrate the diversity of neural response motifs we measured. This includes the diversity of responses to changes in visual orientation and luminance across the cortical population (Figure 1). Additionally, we have added example V1 responses for changes in contrast, spatial frequency, and temporal frequency in order to illustrate the ways in which the joint encoding of these stimulus variables by V1 differs from the encoding of orientation and luminance (Figure 6; Extended Data Fig 6-1).

The reviewers raised important questions about the nature of the V1 code for luminance. In the original submission, we state only that luminance is not represented by a one-dimensional gain change in response across the V1 population. In the resubmitted manuscript, we have revised the Results and Discussion sections to elaborate on our findings regarding how the luminance of visual scenes is represented by the V1 population. In doing so we emphasize that the effect of changing luminance on the V1 population response is high dimensional, with heterogeneous effects across neurons, rather than a one-dimensional gain change of responses. We have added example data to illustrate the diverse range of V1 tuning properties that underlie the population-level representation of luminance. Further details on this finding are given in the responses which follow.

Major Concerns:

1) Almost all the figures present results of analysis while showing very little or none of the original experiments or imaging. Thus, it is difficult as a reviewer to assess the original data and whether the results of the analysis hold up. It would be nice to see some of the raw data in the paper (e.g. what would the mean Ca2+ signals from 1000+ cells look like when plotted for different luminance levels? While the decoder can determine what the luminance level is, is it also obvious to the human eye and would we be able to detect what the pattern is?) Thank you for this suggestion. We recognize the presentation was abstract, and including examples allows us to show more clearly what the data look like and how we are performing our analyses.

We have added examples of Ca2+ fluorescent traces from our V1 dataset throughout the manuscript to show the diversity of tuning properties for simultaneously recorded V1 neurons. We now start the data presentation with a new Figure 1 showing the different types of responses we observe in V1 as we change luminance and orientation. We show traces from distinct V1 subpopulations responding to changes in orientation and luminance. The "most informative orientation cells" have a large change in response magnitude for a change in orientation, which is invariant to scotopic versus photopic luminance. Whereas "most informative luminance cells" exhibit a substantial shift in response magnitude with a change in luminance. The "uninformative cells" in this case are those which show no change in response magnitude for a change in orientation nor luminance.

In Figure 6, we have added example fluorescent traces to show the responses of mouse V1 neurons to drifting gratings at across contrasts, spatial frequencies, and temporal frequencies. The traces in Figure 6A emphasize the contrast-invariance of orientation tuning, which results in orthogonal dU axes for orientation and contrast. The traces in Figure 6B show how orientation tuning varies with spatial frequency in mouse V1, resulting in dU axes for orientation and spatial frequency discrimination that are not orthogonal. Figure 6C shows example V1 neurons tuned to particular combinations of spatial and temporal frequencies corresponding to a particular velocity of drifting grating, resulting in dU axes for temporal and spatial frequency discrimination that are not orthogonal.

2) The methodology to derive discriminability index is a bit opaque. To make the findings more accessible to a wider audience, a more detailed explanation of the rationale behind the quantitative methods, as well as a clearer description of how the dU and e1 components were computed is needed. For instance, the determination of dU (first axis) makes pretty good sense - the difference in response across all trials in different luminance conditions. However, the second axis is hard to decipher. The first principal component of all zero-centered (z-scoresd?) responses is determined (e1), but "The second axis in the decoding space is the component of e1 which is orthogonal to dU- the noise axis." It is unclear what this means nor why the first principal component was not used. Furthermore, the equation for d' is shown and dU is defined but not dU' (is this the orthogonal?), and E is defined as "the 2x2 covariance matrix." Which covariance matrix? We have revised the methods section to describe the dimensionality reduction method used in greater detail. Our explanation echoes that of Heller and David, 2022, who devised this "targeted dimensionality reduction" method. The revised explanation is reproduced below: "To explore what sensory information is present in the high-dimensional V1 population code, we project the V1 population response across stimulus conditions into a two-dimensional space for computing the discriminability index (d'). We employ the "targeted dimensionality reduction" method for projecting neural population responses into an orthonormal basis which preserves the principal axis along which the population response shifts for a change in stimulus (dU) and the axis of the largest component of trial-to-trial covariance in the population response (n1) (Heller and David, 2022).

For each orientation of the grating stimulus, we create two matrices of size Ncells x Ntrials, containing the single trial responses for all simultaneously recorded cells at scotopic and photopic luminance. The difference between the trial-averaged means of these matrices gives the first axis in the decoding space- dU.

Next, we subtract the trial-averaged response for each stimulus condition from the response matrices and concatenate this zero-centered data into a "noise matrix" of size Ncells x 2*Ntrials. We then compute the first principal component of this noise matrix (e1), which represents the primary axis of trial-to-trial covariance in the V1 population response across stimulus conditions.

The axes dU and e1 are not necessarily orthogonal to one another. To form an orthonormal basis for dimensionality reduction, we compute the "noise axis" (n1) as the component of e1 which is orthogonal to dU. Neural data is then projected into the two-dimensional decoding space defined by the orthogonal axes dU and n1. The neural responses for each condition are now represented by a matrix of size 2 x Ntrials. In this two-dimensional space, we compute the difference in mean response to the two luminance conditions (∆U) and the covariance across trials (Σ). The discriminability index (d') is computed as d^'=√(∆U*Σ*∆U') To validate the generalizability of this decoding subspace for capturing the structure of the V1 population response, we hold out a random subset of response trials when fitting the space, and compute d' on the projection of this held out data into the decoding subspace. Each grating stimulus was presented for 50 trials. To fit the decoding subspace, we select neural responses from a random set of 40 trials for each stimulus, and use the remaining 10 trials to compute d'. We repeat this procedure for 50 iterations and compute the average d' across all iterations, which we report as the d' value in our results.

An analogous procedure was used to decode luminance from V1 population responses to natural scenes. We compute each cell's response on a single trial basis as the mean ∆F/F in 166ms bins. Given the slow dynamics of the calcium indicator, this allows us to compare responses to every 5 frames of the natural movies. With these single trial responses across luminance levels, we perform the same luminance decoding procedure as above. " 3) Fig. 1 shows a schematic of the recording paradigm and a schematic of how d' was determined from dU and e1, however, the axes in the second and third plot in Fig. 1B are not labeled and the transformation performed between each is unclear. To the point above including a schematic of how raw responses are translated into dU and e1 would be helpful, as well. Since Fig. 1 jumps straight to a population distribution of the discriminability index, there are no representative examples nor averaged responses of actual neuronal data. It would help immensely to show what type of response variability was observed on at least a few representative neurons. i.e. ones that changed firing in response to luminance but not orientation and vice-versa.

As discussed in Major Concern (1), we have added examples of raw data to Figure 1B. We have also reformatted the illustration in Figure 2A to more clearly show the procedure for decoding visual stimulus information from the V1 population response.

4) Each of the distributions in Fig. 1C and D seem to have two (or more peaks). Are these meaningful (i.e. different populations?). The focus here is on discriminability for luminance, but what about the distribution of neurons based on orientation discriminability? Or a more simple measure such as orientation selectivity? We apologize for the confusion. To clarify, each data point in the histogram from the new Figure 2B (formerly Figure 1C) indicates the d' metric for discriminating luminance level of a grating at one orientation from the V1 population response. Therefore, some of the variability in this histogram is due to diversity in both visual stimuli and experimental population size. We have added Extended Data Fig 2-1A which shows that d' for discriminating luminance is significantly higher for gratings at 100% contrast (mean d' = 5.1 +/- 1.9) than at 30% contrast (mean d' = 3.6 +/- 1.8) (p=.002, two-sample t-test). This stands to reason since higher contrast gratings generally evoke stronger responses in V1 neurons, increasing the signal-to-noise ratio of the V1 population code. While we cannot parametrize distinct frames in the natural scenes presented in Fig 2C in the same way, it is clear that some frames will drive the V1 population more strongly than others, modulating the signal-to-noise ratio of the population response in an analogous manner.

In Extended Data Fig 2-1B we show that d' for luminance discrimination scales with neural population size. There is a clear correlation between the number of simultaneously recorded neurons in an experimental sample and the d' value averaged across stimuli (Pearson's correlation =.68). This is in line with prior results showing a scaling in the fidelity of sensory encoding with neural population size (Kafashan et al., 2021; Stringer et al., 2021).

We have noted these sources of variability in the d' metric in the results section, following the presentation of the results in Figure 2.

5) In Fig. 5, the authors present an argument that luminance encoding in V1 is not mediated by inheriting gain from the retina. Again, presentation of actual responses of luminance informative cells vs. overlapping vs. orientation informative cells would be useful here. In addition, discussion (or even better, exploration) of an alternative model for luminance encoding by V1 would substantially strengthen the manuscript.

As shown in the fluorescent responses plotted in Figure 1B and the scatter plots in Figure 4, V1 neurons have a diversity of response properties for a change in luminance. Some cells have invariant response magnitude with luminance, others have higher responses to scotopic versus photopic luminance, and vice versa. We have updated the Results and Discussion sections to emphasize that the effect of changing luminance on the V1 population response is high-dimensional, or heterogenous across neurons. We have also added an additional modeling example to in Extended Data Fig 7-1, in which we generate modeled V1 fluorescent traces which vary only by a multiplicative gain factor between scotopic and photopic conditions. We contrast these modeled responses with real V1 fluorescent traces to emphasize the heterogeneous effects of luminance across the V1 population. We also emphasize how the joint representation of orientation and luminance differs from that of orientation and contrast, which are also represented along orthogonal axes in the population response, but via a largely unidirectional change in response magnitude with changing contrast.

In the Discussion section, we present a hypothesis for the mechanistic basis of this V1 luminance representation, which relies on projections from ipRGCs with diverse tuning properties. More details on this hypothesis are discussed in our response Minor Concern (5), below.

Minor Concerns:

1) Figure 1: The font size is so small in Fig. 1B as to be nearly illegible. Thus, we cannot really see what the x- and y-labels are in the dDR plot for decoding orientation and in the LDA (? Hard to read) plot (natural scenes).

Thank you for this suggestion. We have resized this portion of that figure, the new Figure 2A, to improve legibility.

2) Figure 3: This plot supports the finding that V1 cells with a strong encoding for orientation were those which encoded orientation and luminance along orthogonal dimensions in the neural response space. However, the color scheme in Fig. 3 makes it hard to discern which cells belong to which group. The plot might be more easily interpretable by indicating there are blue dots for orientation selective only cells (near 0 in du-luminance) and pink dots for luminance selective only cells (near 0 in du-ori), and these are in difference places. However, the proximity of large numbers of black dots (presumably not informative for either) and some number of gray dots at the periphery of the black cloud) make it harder to tell what's going on in the figure. There seems also to be some green (both) data, but the color contrast is not easy for me to tell. Are there gray dots or is this overlap of other categories, could the legend explicitly state the black is nonselective? We have revised this figure, now Figure 4, to make the scatter points belonging to each subpopulation more legible. The color scheme for distinguishing the cell categories has 4 colors: blue, red, green, and black. We did not intend for any scatter points to appear gray, this was an unintended consequence of the marker styles and line weights used in generating the plots. By using larger, filled-in scatter points, the different categories represented in the scatter plots now appear more clearly.

3) In the description of the decoding calculation, line 464-465 mentions "held-out" trials projected into decoding space. Which trials are these? Why were all data not included? As discussed above in Major Concern (2), we have added a more complete description of our dimensionality reduction procedure to the methods section: "To validate the generalizability of this decoding subspace for capturing the structure of the V1 population response, we hold out a random subset of response trials when fitting the space, and compute d' on the projection of this held out data into the decoding subspace. Each grating stimulus was presented for 50 trials. To fit the decoding subspace, we select neural responses from a random set of 40 trials for each stimulus, and use the remaining 10 trials to compute d'. We repeat this procedure for 100 iterations and compute the average d' across all iterations, which we report as the d' value in our results. " 4) The rationale behind the choices of orientations shown is not described. For drifting gratings a typical 45deg spacing is used, but for static gratings a somewhat random array is used. If this is so comparisons between 10deg and 45deg differences can be compared, this is not explicitly stated anywhere. The only mention present was analysis of 45deg changes in calculating orientation (line 177). Why not use something more informative like an orientation-selectivity index? Thank you for pointing out the lack of clarity on this point. We have updated the methods section to clarify the different grating stimulus sets used throughout our experiments: "In experiments comparing V1 responses under scotopic and photopic luminance conditions, static sinewave gratings were presented at .05 cycles/{degree sign} for 500ms at 6 orientations (0, 10, 45,90, 100,135{degree sign}) and 2 contrasts (30%, 100%), each followed by a 1 second blank screen at mean luminance. We presented gratings differing by only 10{degree sign} in order to probe how small changes in orientation are encoded by the V1 population across luminance levels. Specifically, we tested whether the V1 population encoded this small orientation difference with equal fidelity across scotopic and photopic luminance levels. The results of this analysis have been presented previously, showing that the d' for ∆10{degree sign} has equal magnitude between scotopic and photopic luminance (O'Shea et al., 2025). For the analysis presented in Figure 2 and Figure 7 in which we probed the V1 representation of mean luminance in response to otherwise identical stimuli, we compared the V1 population response to gratings at each of these 6 orientations and 2 contrasts, differing only in mean luminance. For the analyses in Figures 4-5, in which we ask how the V1 population encodes changes in orientation along with changes in luminance, only responses to pairs of gratings separated by 45{degree sign} were considered. For the analysis in Figure 6A, in which we ask how the V1 population encodes changes in orientation along with changes in contrast, only responses to pairs of gratings separated by 45{degree sign} were considered.

In the experiments presented in Fig 6B, drifting sinewave gratings were presented for 2000ms at 8 orientations (0, 45, 90,135,180, 225, 270, 315{degree sign}) and 5 spatial frequencies (.02, .04, .08, .16, .32 cycles/degree), each followed by a 1 second blank screen at mean luminance.

In the experiments presented in Fig 6C, drifting sinewave gratings were presented for 2000ms at 1 orientation (0{degree sign}), 5 spatial frequencies (.02, .04, .08, .16, .32 cycles/degree), and 4 temporal frequencies (1, 2, 4, 8 Hz), each followed by a 1 second blank screen at mean luminance." 5) How does this vary across species? It would be nice to indicate if this has been tested in other animals (e.g. how does a human know luminance level? I feel when I read indoors and outdoors that the page looks largely the same, even though (a) the photon flux is much much (several orders of magnitude) lower indoors, and (b) Even though it most looks the same ... there is some awareness that it's brighter or dimmer in the environment ... how do our brains do that? Is there some proposed circuit mechanism? Or a simplified idea of how the detector can tell what the luminance level is, and does this vary across humans/mice? To address this point, we have added information about the known results on luminance encoding in V1 across species to the Discussion section. We have also elaborated on our hypothesis that projections from the ipRGCs to central visual areas could provide and explicit representation of luminance. Our additions are reproduced in condensed form, below:

The luminance-invariant V1 orientation and spatial frequency tuning documented in mouse have also been shown in macaque V1 (Duffy and Hubel, 2007; O'Shea et al., 2025). Human psychophysical experiments have shown that the perceptual attenuation of spatial and temporal contrast sensitivity for low frequencies, first measured under photopic conditions in human subjects, persists in scotopic conditions. In addition, the perceptual phenomenon of simultaneous contrast, in which squares of equal luminance appear to differ in brightness depending on the luminance of the surrounding area, persists in the scotopic regime (Fiorentini and Maffei, 1973). These results indicate that luminance-invariant spatial encoding is a general property of mammalian V1, and raises the question of how luminance could be represented by the visual system.

Psychophysical experiments indicate that humans are aware of slow changes in mean luminance of the visual scene despite luminance adaptation in the retina and the luminance-invariant response properties of downstream neurons. These results suggest that an explicit signal for luminance, perhaps originating in the ipRGCs, provides a representation of luminance to downstream visual areas. Indeed, ipRGCs project to the lateral geniculate nucleus of the thalamus (LGN) in both mouse and primate, implicating this class of neurons in the thalamocortical visual circuit (Dacey et al., 2005; Brown et al., 2010). ipRGCs have overlapping spectral sensitivity with mouse rod and M-cone photoreceptors, and will therefore be differentially activated by the 525 nm scotopic and photopic stimuli used in this study. Humans and mice can behaviorally discriminate the brightness of visual metamers that do not alter rod or cone activation, but do differentially activate melanopsin, the photopigment present in ipRGCs (Brown et al., 2012). Knockout mice lacking rod and cone photoreceptors can still perform a visually guided task in which they must discriminate the brightness of two displays in order to exit a water maze (Brown et al., 2012). These findings suggest that signals from ipRGCs projecting to the LGN could modulate the V1 population response across light levels.

In this study we have shown that the V1 representation of mean luminance is not due to a one-dimensional shift in the gain of the population response. Rather, changing luminance has diverse effects on V1 neurons, with some neurons showing no change in visually-evoked response magnitudes between luminance conditions, others having higher response magnitudes in response to scotopic relative to photopic stimuli, and still others responding more strongly in the photopic than scotopic condition. Our observation that luminance can modulate V1 responses bidirectionally may be at odds with the canonical finding that ipRGCs respond monotonically to increases in luminance (Do and Yau, 2010). Any proposed circuit mechanism for the encoding of luminance in V1 must account for this bidirectional modulation of V1 responses across the population, given the monotonic modulation of ipRGC firing rate by luminance. Interestingly, recent studies of ipRGCs in the mouse retina have identified functional clusters of ipRGCs which have distinct dynamic ranges for encoding luminance (Sondereker et al., 2020). Some classes of ipRGCs are more strongly driven at scotopic than photopic luminance, breaking from the canonical view of ipRGCs as monotonic encoders of luminance. A possible explanation for the diverse effects of luminance on V1 responses could be that projections from these functional clusters of ipRGCs remain segregated in downstream visual areas.

6) At very low luminance (I'm gathering lower than attempted here), the ability to detect anything at all must go to chance, so is there some threshold level which must be reached to have luminance invariance of orientation selectivity? O'Shea et al. 2025 found luminance-invariant receptive field properties in mouse V1 within the .016 - 97 cd/m2 range, corresponding to a moonlit night in a rural setting and a summer day in direct sunlight (Spitschan et al., 2016; Rhim et al., 2021). The study did not examine at what luminance level this invariance breaks down. Based on prior studies, we predict that this invariance breaks down at luminance levels below those encountered by mammals in the terrestrial environment.

The demands on the visual system fundamentally shift at ultra-low light levels (below 10-3 cd/m2), with arrival of photons at the retina being so sparse that a shift in processing toward simple signal detection, via lowpass filtering of the rod-mediated signal, is a more efficient strategy than attempting to extract higher frequency spatiotemporal features. This is because there may be insufficient illumination to discern these features, such that bandpass filtering would only amplify higher frequency noise in phototransduction from the rods (Atick and Redlich, 1990; Field et al., 2005). The retinal circuitry by which rod signals are relayed to the retinal ganglion cells shifts at this ultra-low luminance, with only the rod bipolar cells (the "primary" rod pathway) being active. At the higher end of the scotopic range, as used in our experiments, a portion of the rod output is relayed through gap junctions to the cones and onto the RGCs through the same circuitry which relays cone-mediated signals at higher luminance levels. This circuitry propagates signals from the photoreceptors with shorter integration times and spatial receptive fields tuned to higher spatial frequencies than the primary rod pathway (Field and Chichilnisky, 2007; Grimes et al., 2018). In keeping with these distinct circuit mechanisms operating within the scotopic range, retinal receptive fields shift substantially across the scotopic range (Mastronarde, 1983; Greschner et al., 2011; Ruda et al., 2022). In mice, the contrast sensitivity of the reflexive optomotor response to drifting gratings retains invariant, bandpass spatial frequency tuning across the range of scotopic and photopic luminance employed in our study, but shifts toward lower spatial and temporal frequencies at 10-4.5 cd/m2 and below, approaching the threshold of rod activation (10-6 cd/m2) (Umino et al., 2008). This result likely reflects a downstream consequence of changing retinal function over the scotopic range.

7) Line 200+: "To test this idea, we computed decoding axes for V1 population responses to 45{degree sign} changes in the orientation of drifting gratings, and to 1 octave changes in the SF of gratings at a fixed orientation." Could this data be plotted as in Fig 3.? It seems weird to not show how the results look different when plotted for a different pair of parameters to decode but instead plot more derived features in Fig. 4). This also applies to the temporal frequency.

Thank you for this suggestion. Yes, this was an oversight. We have added example fluorescent traces to Figure 6 to illustrate the dependencies between the coding for orientation and contrast, orientation and spatial frequency, and spatial and temporal frequency. We have also added Extended Data Fig 6-1, showing example scatter plots of the decoding weights for each of these visual stimulus features. These example plots allow for comparison to the decoding weights for orientation versus luminance presented in Figure 4.

8) Line 218+: If the "most informative" neurons for orientation or luminance discrimination are "those with the top 10% dm magnitude, or the largest trial-averaged change in response as a function of a change in stimulus", then why isn't the line separating the red/blue/black data in Fig. 3 more straight? Or maybe it is straight and the gray dots make it hard to tell? As discussed above in Minor Concern (2), we have revised the formatting of the scatter plots in the new Figure 4 to make the scatter points more legible. The separation between each color-coded category now appears straighter.

9) Figure 5 explores whether a linear decoder can separate responses from two luminance conditions assuming the effect on firing is simply a change in gain. This seems to be performed with modeled data? Perhaps I misunderstand (Line 255: "if we normalize the modeled responses").

Thank you for pointing out the lack of clarity on this. To clarify, the analysis in the new Figure 7 (formerly Figure 5) is performed first on modeled V1 responses generated to simulate a one-dimensional gain change in the V1 population response across luminance levels. We then perform the same analysis on the real V1 data in order to show that the luminance representation in V1 is not due to a one-dimensional gain shift. This result emphasizes that a change in luminance has a diverse range of effects on V1 responses across the neural population. We elaborate further on the reasoning behind this analysis, below. We have also elaborated on this modeling exercise in the Methods section.

If the only change in the V1 population response between luminance conditions were a one-dimensional gain shift, then normalizing responses to the maximum response within each condition, to equalize response magnitude, would eliminate the V1 representation of luminance. To illustrate this point, we present a simple modeling exercise in Fig 7a-b, in which we simulate the responses of 2 model neurons to repeated presentations of a stimulus at scotopic and photopic luminance. The modeled responses are drawn from a multivariate normal distribution using "mvnrnd" function in MATLAB. To simulate a shift in the gain of V1 responses between scotopic and photopic conditions, we scale the mean and variance of the multivariate normal distribution. The distribution of modeled responses is shown in the left panel of Fig 7a. We then perform an identical dimensionality reduction and decoding procedure as for our real neural data to compute the d' metric, as illustrated in the right panel of Fig 7a. Next, we normalize the modeled responses within each condition. In Fig 7b we show that this normalization procedure makes the distribution of modeled responses for the two luminance levels indistinguishable for our decoder, with d' declining to chance values.

This modeling exercise establishes that the response normalization procedure eliminated the representation of luminance in our modeled V1 population, for which luminance level was represented by a one-dimensional shift in the gain of V1 responses. We then applied an identical normalization procedure to our real neural data, and found that the V1 luminance representation persisted, as d' values were unchanged. In Fig 7c we plot the d' values for each experiment before and after normalization, with all points lying along unity, indicating that the normalization procedure did not reduce the discriminability of the V1 population code for luminance. This result emphasizes that the V1 population representation of luminance is due to heterogenous shifts in response magnitude across the V1 population.

10) Line 279: "We have shown that the mouse V1 population encodes mean luminance and orientation in a separable manner." Can you give some insight into the circuit or synaptic mechanism? Do we understand why these are separable? Is it just the separate cell populations encode each (e.g., let's take the orientation selective ones as one population, and luminance selective as separate groups that just happen to be spatially intermingled)? Are these just a random subset of the whole neuron population? (e.g. we could do it with 10% for luminance and 10% for orientation and the other 80% are set aside for ... other orientations or characteristics?) Are they spatially intermingled in other mammals (cat, primate)? These are all good questions. Our results indicate that the representation of luminance is distributed across heterogeneous shifts in the responses of V1 neurons to a change in luminance. In the revised Results section, we emphasize our finding that the distribution of response motifs is analogous to the encoding of orientation by the V1 population, in that a change in the stimulus shifts neural responses in a bidirectional manner, or can have no effect on response magnitude, depending on the tuning properties of each neuron. We have also added to the results section and Figure 4 to show that we do not observe any spatial organization of the neurons that are most informative for orientation or luminance. Rather, these populations are intermingled across the imaging plane. We hypothesize in the Discussion section that diverse projection patterns of ipRGCs to central visual areas could underlie the distribution of responses which we measured in V1.

Importantly, we found that the neurons which had the largest change in response for a given change in orientation (d-ori) were distinct from the neurons which were most modulated by a change in luminance (d-luminance). As we show in Figure 3, a consequence of this segregation, in which there was no correlation between d-ori and d-luminance, is that the V1 population encodes these two stimulus variables along orthogonal dimensions. These representations are therefore separable, such that a luminance-invariant spatial representation of visual features coexists with a flexible representation of luminance level.

To our knowledge, there has been no population-scale analysis comparing the functional properties of V1 neurons at scotopic and photopic luminance in higher mammals. It is unknown how neurons encoding changes in orientation and luminance are distributed in V1 of cats or primates.

11) How might downstream visual circuits extract mean luminance? One suggestion is a "functional shift" in the "spatiotemporal or chromatic responses of neurons in central visual areas". Wouldn't cat/human be better to study this question since the central-to-peripheral gradient is somewhat steeper in the retina of these animals? We agree that these are useful questions to discuss in the paper. We have added to the Discussion section to address potential differences and commonalities in the V1 representation of luminance across the species. Our additions are reproduced in condensed form, below: "Across species, the chromatic tuning of the retinal ganglion cells shifts from scotopic to photopic luminance due to the shift from rod to cone-mediated phototransduction (Baylor et al., 1987). This shift in chromatic tuning at the sensory periphery alters the chromatic tuning of cortical neurons (Rhim et al., 2017, 2021; Rhim and Nauhaus, 2023). Therefore, across mammalian species, a shift in the chromatic tuning of cortical neurons could provide an implicit representation of mean luminance.

It is possible that luminance adaptation state is encoded implicitly via functional shifts in the spatiotemporal or chromatic responses of neurons in central visual areas. Functional shifts which are reliably tied to a change in mean luminance provide information about the mean luminance of the visual input. For example, species with a rod-free fovea, such as primates, lose visual sensitivity at the center of the visual field when adapted to luminance below the threshold for cone activation. This functional shift in the output of the retina alters the visual information available to downstream circuitry. In primate, a simple representation of luminance adaptation state could be whether or not V1 neurons with receptive fields within the foveal retinotopy are visually driven. Importantly, outside of the foveal zone, the spatial selectivity of macaque V1 cells is largely invariant across scotopic and photopic conditions, in line with recent findings in the mouse (Duffy and Hubel, 2007; O'Shea et al., 2025). Furthermore, human psychophysical experiments have shown that the perceptual attenuation of spatial and temporal contrast sensitivity for low frequencies, first measured under photopic conditions in human subjects, persists in scotopic conditions. In addition, the perceptual phenomenon of simultaneous contrast, in which squares of equal luminance appear to differ in brightness depending on the luminance of the surrounding area, persists in the scotopic regime (Fiorentini and Maffei, 1973). These results indicate that luminance-invariant spatial encoding is a general property of mammalian V1, and raises the question of how luminance could be represented by V1 neurons in the absence of changes in receptive field structure. In this study, we find that changes in mean luminance are encoded by diverse changes in V1 neural responses despite luminance adaptation in the retina and the luminance-invariant tuning properties of V1 neurons." 12) ~Line 314: The result "We found, however, that normalizing V1 response magnitude across light levels did not alter the ability to decode luminance state suggesting that additional signals are present in V1 that are related to luminance state" is somewhat not fully satisfying since it suggests that firing rate is not needed to decode luminance but leaves unanswered perhaps what does suffice? The ipRGC hypothesis is interesting but could be tested.

In the resubmitted manuscript, we have revised the Results and Discussion sections to elaborate on our findings regarding how the luminance of visual scenes is represented by the V1 population. In doing so we emphasize that the effect of changing luminance on the V1 population response is high dimensional, with heterogeneous effects across neurons, rather than a one-dimensional shift in the gain of responses. We present representative fluorescent traces from simultaneously recorded neurons driven by gratings at scotopic and photopic luminance. In examining these examples, the diversity of response changes with a change in luminance is apparent. We tie these observations to the scatter plots for d-ori and d-luminance in Figure 4 to emphasize that changing luminance has heterogeneous, bidirectional effects on responses across the V1 population.

We have added example data in Figure 1 and Extended Data Fig 7-1 to illustrate the diverse range of V1 tuning properties that underlie the population-level representation of luminance. The change in luminance is, of course, encoded by changes in the firing rates of neurons. Our analysis in Figure 7, in which we normalize responses to the maximum within each luminance condition, shows that luminance is not encoded by a uniform shift in response magnitude across the V1 population. Rather, changing luminance has diverse effects on V1 activity, with some cells having invariant responses across luminance, others having higher responses to scotopic versus photopic luminance, and vice versa. This distribution of functional properties in the V1 population is apparent in the scatter plots for d-ori and d-luminance. Our results indicate that the representation of luminance is distributed across diverse changes in the firing rates of V1 neurons. In the Discussion section, we hypothesize about how projections from ipRGCs could underlie this sort of population code. This is an interesting future direction for this line of work.

We also emphasize how the joint encoding of orientation and luminance differs from that of other stimulus variables. The joint representation of orientation and luminance is distinct from that of orientation and contrast, which are also encoded along orthogonal axes by the V1 population, in that a change in luminance has diverse, bidirectional effects on response magnitudes across the V1 population. The encoding of orientation and luminance is also distinct from the encoding of other visual properties, such as spatial and temporal frequency, which are not encoded independently by the V1 population.

Works Cited Atick JJ, Redlich AN (1990) Towards a Theory of Early Visual Processing. Neural Computation 2:308-320.

Baylor DA, Nunn BJ, Schnapf JL (1987) Spectral sensitivity of cones of the monkey Macaca fascicularis. J Physiol 390:145-160.

Brown TM, Gias C, Hatori M, Keding SR, Semo M, Coffey PJ, Gigg J, Piggins HD, Panda S, Lucas RJ (2010) Melanopsin Contributions to Irradiance Coding in the Thalamo-Cortical Visual System. PLOS Biology 8:e1000558.

Brown TM, Tsujimura S, Allen AE, Wynne J, Bedford R, Vickery G, Vugler A, Lucas RJ (2012) Melanopsin-Based Brightness Discrimination in Mice and Humans. Current Biology 22:1134-1141.

Dacey DM, Liao H-W, Peterson BB, Robinson FR, Smith VC, Pokorny J, Yau K-W, Gamlin PD (2005) Melanopsin-expressing ganglion cells in primate retina signal colour and irradiance and project to the LGN. Nature 433:749-754.

Do MTH, Yau K-W (2010) Intrinsically photosensitive retinal ganglion cells. Physiol Rev 90:1547-1581.

Duffy KR, Hubel DH (2007) Receptive field properties of neurons in the primary visual cortex under photopic and scotopic lighting conditions. Vision Research 47:2569-2574.

Field GD, Chichilnisky EJ (2007) Information processing in the primate retina: circuitry and coding. Annu Rev Neurosci 30:1-30.

Field GD, Sampath AP, Rieke F (2005) RETINAL PROCESSING NEAR ABSOLUTE THRESHOLD: From Behavior to Mechanism. Annual Review of Physiology 67:491-514.

Fiorentini A, Maffei L (1973) Contrast in night vision. Vision Research 13:73-80.

Greschner M, Shlens J, Bakolitsa C, Field GD, Gauthier JL, Jepson LH, Sher A, Litke AM, Chichilnisky EJ (2011) Correlated firing among major ganglion cell types in primate retina. J Physiol 589:75-86.

Grimes WN, Baudin J, Azevedo AW, Rieke F (n.d.) Range, routing and kinetics of rod signaling in primate retina. eLife 7:e38281.

Heller CR, David SV (2022) Targeted dimensionality reduction enables reliable estimation of neural population coding accuracy from trial-limited data. PLOS ONE 17:e0271136.

Kafashan M, Jaffe AW, Chettih SN, Nogueira R, Arandia-Romero I, Harvey CD, Moreno-Bote R, Drugowitsch J (2021) Scaling of sensory information in large neural populations shows signatures of information-limiting correlations. Nat Commun 12:473.

Mastronarde DN (1983) Correlated firing of cat retinal ganglion cells. II. Responses of X- and Y-cells to single quantal events. J Neurophysiol 49:325-349.

O'Shea RT, Nauhaus I, Wei X-X, Priebe NJ (2025) Luminance invariant encoding in mouse primary visual cortex. Cell Reports 44 Available at: https://www.cell.com/cell-reports/abstract/S2211-1247(24)01568-7 [Accessed March 17, 2025].

Rhim I, Coello-Reyes G, Ko H-K, Nauhaus I (2017) Maps of cone opsin input to mouse V1 and higher visual areas. J Neurophysiol 117:1674-1682.

Rhim I, Coello-Reyes G, Nauhaus I (2021) Variations in photoreceptor throughput to mouse visual cortex and the unique effects on tuning. Sci Rep 11:11937.

Rhim I, Nauhaus I (2023) Joint representations of color and form in mouse visual cortex described by random pooling from rods and cones. J Neurophysiol 129:619-634.

Ruda K, Rudzite AM, Field GD (2022) The functional organization of retinal ganglion cell receptive fields across light levels. :2022.09.15.508164 Available at: https://www.biorxiv.org/content/10.1101/2022.09.15.508164v1 [Accessed September 22, 2023].

Sondereker KB, Stabio ME, Renna JM (2020) Crosstalk: The diversity of melanopsin ganglion cell types has begun to challenge the canonical divide between image-forming and non-image-forming vision. J Comp Neurol 528:2044-2067.

Spitschan M, Aguirre GK, Brainard DH, Sweeney AM (2016) Variation of outdoor illumination as a function of solar elevation and light pollution. Sci Rep 6:26756.

Stringer C, Michaelos M, Tsyboulski D, Lindo SE, Pachitariu M (2021) High-precision coding in visual cortex. Cell 184:2767-2778.e15.

Umino Y, Solessio E, Barlow RB (2008) Speed, Spatial, and Temporal Tuning of Rod and Cone Vision in Mouse. J Neurosci 28:189-198.
