# Supplementary figures and images for: Independent Encoding of Orientation and Mean Luminance by Mouse Visual Cortex
Source: eNeuro. 2026 Feb 6;13(2):ENEURO.0281-25.2025. doi: 10.1523/ENEURO.0281-25.2025 (PMC12893793; doi:10.1523/ENEURO.0281-25.2025)

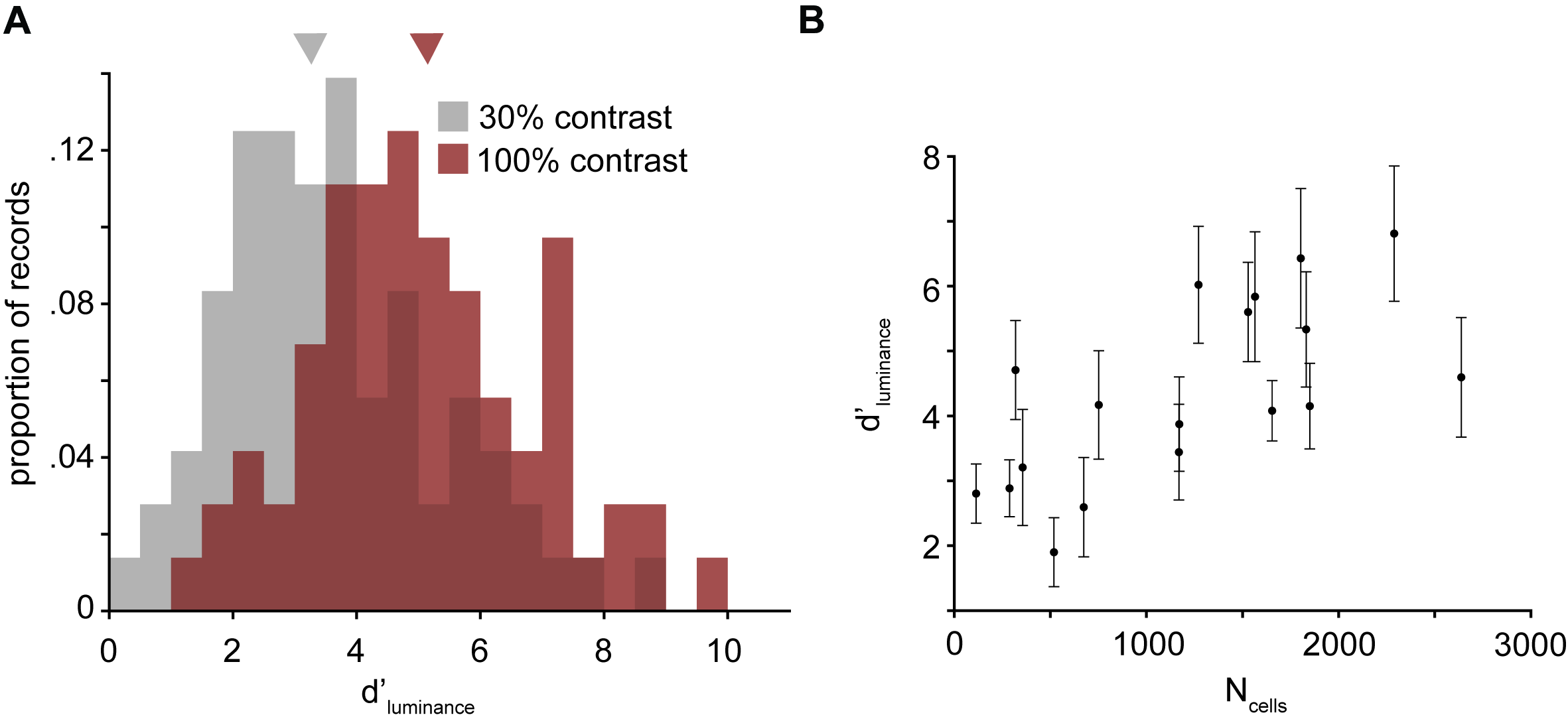

Supplement: Figure 2-1 — Discrimination index for scotopic vs photopic luminance. A. Histogram of d’ values across all V1 imaging experiments for discriminating responses to gratings of the same orientation at scotopic and photopic luminance, plotted separately for 30% and 100% contrast gratings. Arrows indicate mean across all experiments and grating orientations at a single contrast. B. Scatter plot showing mean d'luminance as a function of V1 population size. Black dots indicate mean across experimental stimuli pairs, errorbars indicate +/- .5 standard deviation. Download Figure 2-1, TIF file. [file eneuro-13-ENEURO.0281-25.2025-s001.tif]

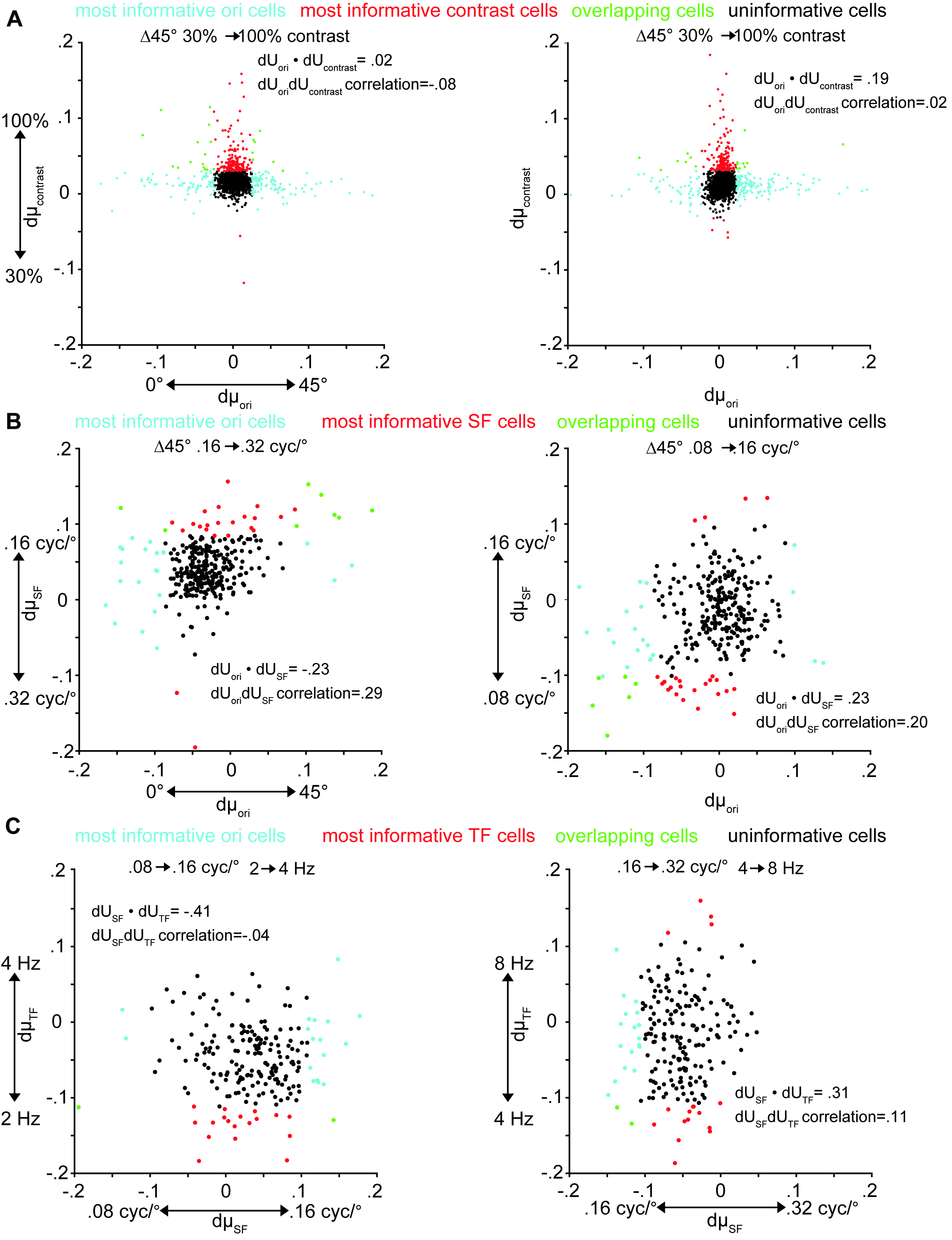

Supplement: Figure 6-1 — Experimental dμ and dμ distributions. A. Example V1 population dμori and dμcontrast distributions. Each scatter point gives the dμori and dμcontrast magnitude for a V1 cell. Top 10% largest dμ magnitude cells are highlighted for orientation (cyan) and contrast (red) discrimination. Cells that overlap in both most informative subpopulations are highlighted in green. B. As in A, for dμori and dμSF. C. As in A, for dμSF and dμTF. Download Figure 6-1, TIF file. [file eneuro-13-ENEURO.0281-25.2025-s002.tif]

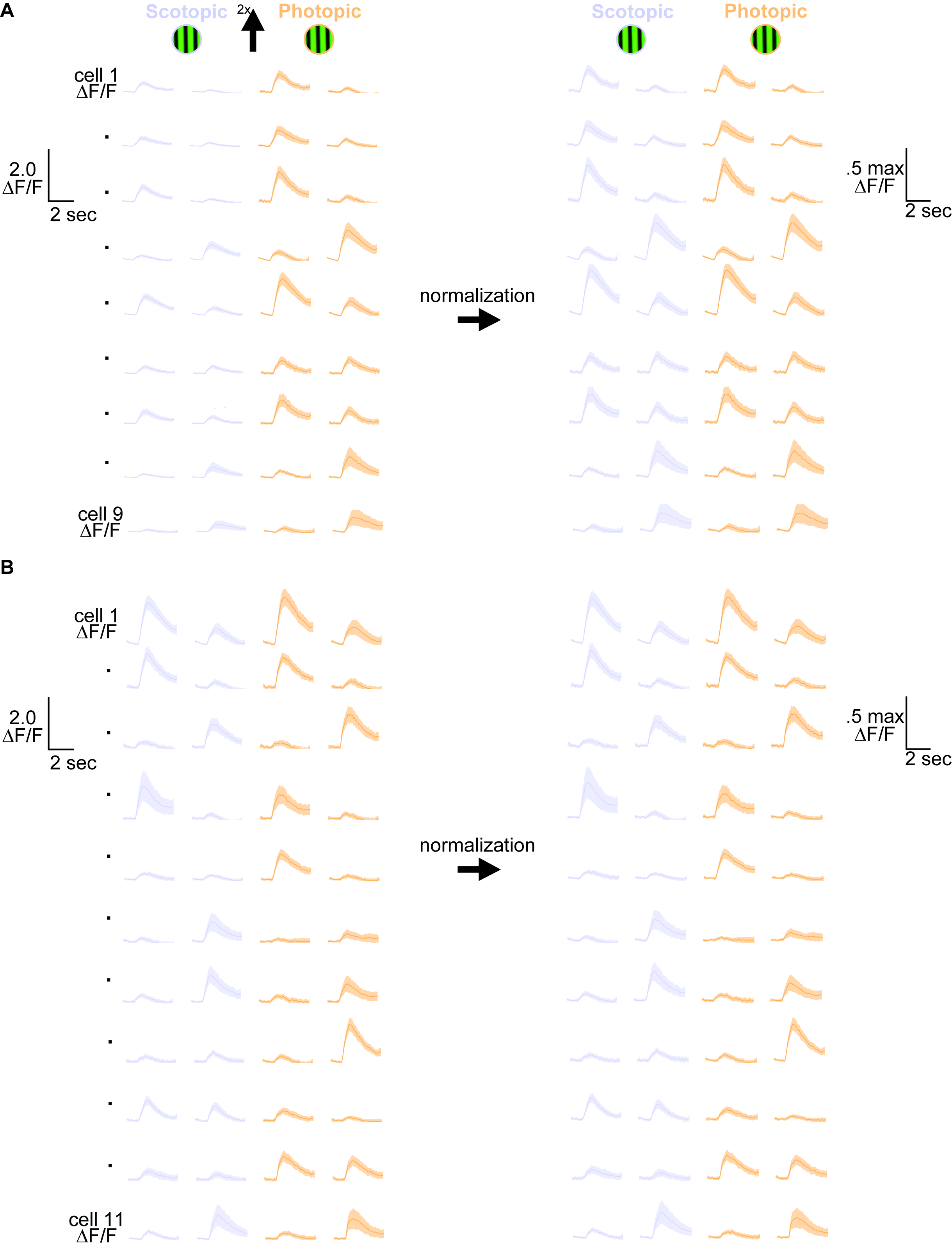

Supplement: Figure 7-1 — Modeling V1 responses to scotopic and photopic luminance. A. (left) Modeled fluorescent traces from V1 neurons responding 0° and 45° gratings at scotopic and photopic luminance. The photopic responses are the scotopic responses scaled by a factor of 2. (right) Modeled fluorescent traces from V1 neurons following normalization to the maximum response across all neurons in each light condition. B. (left) Real fluorescent traces from simultaneously recorded V1 neurons responding 0° and 45° gratings at scotopic and photopic luminance. (right) Real fluorescent traces from V1 neurons following normalization to the maximum response across all neurons in each light condition. Solid line indicates the trial-averaged response, shaded region indicates +/- 1 standard deviation. Download Figure 7-1, TIF file. [file eneuro-13-ENEURO.0281-25.2025-s003.tif]
